# Treatment with mRNA coding for the necroptosis mediator MLKL induces antitumor immunity directed against neo-epitopes

**DOI:** 10.1038/s41467-018-05979-8

**Published:** 2018-08-24

**Authors:** Lien Van Hoecke, Sandra Van Lint, Kenny Roose, Alexander Van Parys, Peter Vandenabeele, Johan Grooten, Jan Tavernier, Stefaan De Koker, Xavier Saelens

**Affiliations:** 1 VIB Center for Medical Biotechnology, VIB, 9000 Ghent, Belgium; 2 Department of Biomedical Molecular Biology, Ghent University, 9000 Ghent, Belgium; 3 Cytokine Receptor Laboratory, Department of Biochemistry, Faculty of Medicine and Health Sciences, Ghent University, 9000 Ghent, Belgium; 4 VIB-UGent Center for Inflammation Research, VIB, 9000 Ghent, Belgium

## Abstract

Cancer immunotherapy can induce durable antitumor responses. However, many patients poorly respond to such therapies. Here we describe a generic antitumor therapy that is based on the intratumor delivery of mRNA that codes for the necroptosis executioner mixed lineage kinase domain-like (MLKL) protein. This intervention stalls primary tumor growth and protects against distal and disseminated tumor formation in syngeneic mouse melanoma and colon carcinoma models. Moreover, MLKL-mRNA treatment combined with immune checkpoint blockade further improves the antitumor activity. MLKL-mRNA treatment rapidly induces T cell responses directed against tumor neo-antigens and requires CD4^+^ and CD8^+^ T cells to prevent tumor growth. Type I interferon signaling and Batf3-dependent dendritic cells are essential for this mRNA treatment to elicit tumor antigen-specific T cell responses. Moreover, MLKL-mRNA treatment blunts the growth of human lymphoma in mice with a reconstituted human adaptive immune system. MLKL-based treatment can thus be exploited as an effective antitumor immunotherapy.

## Abstract (web summary)

Necroptosis has immunogenic cell death properties. Here, the authors show that the intra-tumor delivery of mRNA that codes for the necroptosis effector MLKL triggers neo-epitope-specific anti-tumor T cell responses and inhibits primary tumor growth and lung metastasis.

## Introduction

Cancer cells evade the immune system in many ways. The clinical success of immunotherapies that are based on the (re-)activation of antitumor T cells has revolutionized cancer treatment and highlights the tremendous power of T cells to control malignant diseases^1–3^. Nonetheless, the majority of patients remain unresponsive to the current immunotherapies that are based on so called checkpoint inhibitors^4–6^. A growing body of evidence indicates that checkpoint inhibitor unresponsiveness correlates with a lack of CD8^+^ T cells inside the tumor^6,7^. The extent of T cell infiltration into tumors in turn depends on prior innate immune activation in the tumor microenvironment (TME) and the recruitment of Batf3-dependent CD103^+^ dendritic cells (DCs)^8^. These Batf3-dependent DCs are not only required for the initial priming of antitumor T cell responses in the tumor draining lymph nodes but also secrete the appropriate chemokines to attract effector T cells^8^. Defective T cell priming could potentially be overcome by active vaccination strategies directed against tumor antigens or by adoptive T cell therapies. However, immunologically quiescent tumors can resist such strategies because T cells fail to migrate into the tumor bed^8^.

An immunogenic tumor environment can be created by eliciting immunogenic cell death, which represents a common denominator for a variety of cell death pathways that result in the release of damage-associated molecular patterns (DAMPs) and other immune-stimulatory components that can recruit and activate DCs in the TME^9–11^. For example, immunogenic apoptosis of neoplastic cells has been documented in response to irradiation, chemotherapeutics, and hypericin-based photodynamic therapy^12–16^. In addition to certain apoptosis modalities, necroptosis has been identified as a type of cell death with immunogenic properties^17,18^. Necroptosis can be induced by activation of death receptors, Toll-like receptors, intracellular RNA and DNA sensors, and by some chemical drugs^19^. The core necroptotic pathway involves phosphorylation of receptor interacting protein kinase 3 (RIPK3), which subsequently phosphorylates mixed lineage kinase domain-like protein (MLKL)^20–25^. Phosphorylated MLKL oligomerizes and subsequently translocates to the plasma membrane where it inflicts membrane permeabilization and necroptosis^23–28^. Strikingly, genetic and epigenetic changes in the pathways that lead to necroptosis have been described for many tumor types. Strongly reduced RIPK3 expression levels, the kinase that phosphorylates and thereby activates MLKL, for example, have been documented in colon carcinoma and are frequent in acute myeloid and chronic lymphocytic leukemia^29^. Moreover, in pancreatic cancers, reduced MLKL expression is associated with decreased survival^30,31^.

We hypothesized that genetic delivery of MLKL into the TME could create an immunogenic environment that subsequently instills adaptive antitumor immunity. For this delivery, we opted to apply in vitro transcribed mRNA as a way to express MLKL in the TME because mRNA has emerged as an extremely versatile platform to deliver genetically encoded therapeutics in situ^32,33^. We demonstrate that intratumor administration of mRNA encoding MLKL elicits a potent antitumor T cell response—involving T cells directed against tumor neo-antigens—even in tumors that are defective for upstream necroptotic signaling proteins. MLKL-mRNA treatment protected in two syngeneic mouse tumor models and even in mice with a humanized immune system that had been inoculated with human lymphoma cells.

## Results

### MLKL mRNA induces necroptosis-like tumor cell death

In vitro transcribed mRNA has been widely explored to deliver directly translatable coding information in in vitro cultured cells, in experimental animal models, and in patients^34,35^. We therefore generated hypo-inflammatory mRNAs (Supplementary Fig. 1a-b) to assess the potential antitumor outcome of transiently expressed MLKL and, in comparison, truncated Bcl2-like inducer of cell death (tBid). MLKL is crucial for the execution of necroptosis, while tBid, the caspase-cleaved form of Bid, is an inducer of intrinsic apoptotic cell death^22,36^. First, we assessed the kinetics of mRNA uptake and translation. Fluorescently labeled green fluorescent protein (GFP)-mRNA was rapidly detectable in transfected B16 melanoma cells and the expression of the encoded GFP became visible 8 h after transfection (Supplementary Fig. 1c). In vivo, similar expression kinetics were found: intratumor delivery of mRNA resulted in a peak of expression of the encoded firefly luciferase (Fluc) reporter at 12 h after electroporation of injected mRNA (Supplementary Fig. 1d).

**Fig. 1:**
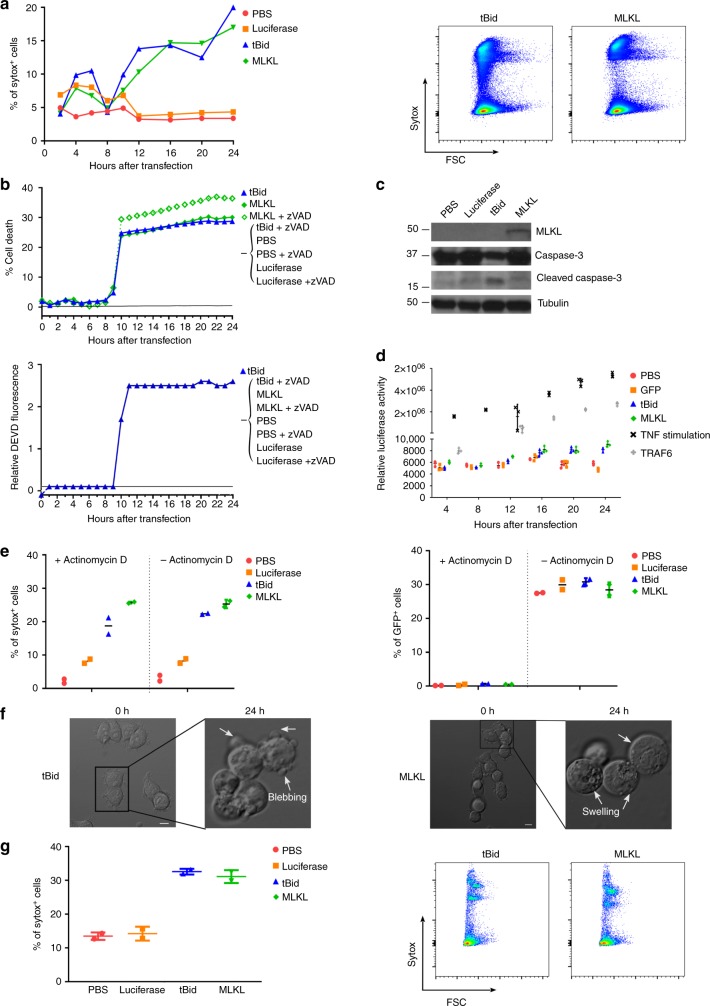
MLKL encoding mRNA induces cell death in vitro and in vivo. **a**–**f** In vitro cell death characterization. B16-OVA cells were transfected with PBS or with Fluc-, tBid-, or MLKL-mRNA. **a** At different time points after transfection, cells were collected and analyzed by flow cytometry. Graph showing the percentages of sytox^+^ cells (left). Representative flow cytometric plots 24 h after transfection (right). **b** Impact of the pan-caspase inhibitor zVAD-fmk on cell death induction (top) and caspase activity (bottom) upon transfection of B16-OVA cells with tBID- or MLKL-mRNA. **c** Western blot analysis of the expression of MLKL, caspase-3, and cleaved caspase-3 in cell lysates prepared 24 h after mRNA transfection. Tubulin served as a loading control. **d** B16 cells were co-transfected with luciferase NF-κB reporter plasmid and a plasmid expressing β-galactosidase. Twenty four hours later, the cells were transfected with PBS, GFP-, tBid-, or MLKL-mRNA or, as a positive control for NF-κB activation, with TRAF6 expression vector or they were stimulated with TNF. The normalized luciferase activity in the lysates determined at different time points after mRNA transfection is depicted. **e** B16 cells were transfected with a GFP expression plasmid. Twenty four hours later, the cells were transfected with PBS, Fluc-, tBid-, or MLKL-mRNA and the cells were treated or not with actinomycin D as indicated. Twenty four hours after mRNA transfection, cell death and GFP expression were quantified using flow cytometry. Horizontal lines indicate the mean. **f** Still images of B16-OVA cells at 0 and 24 h after transfection with tBid- or MLKL-mRNA visualized by time-lapse microscopy (scale bar = 10 µm). **g** In vivo cell death characterization. Subcutaneously growing B16-OVA tumors were injected with PBS or with 10 µg mRNA encoding Fluc, tBid, or MLKL followed by electroporation. Twenty four hours later, the tumor cells were isolated and analyzed by flow cytometry for sytox uptake. Graph showing the percentages of sytox^+^ cells (left) and representative flow cytometry plots (right). Horizontal lines indicate the mean

We next analyzed the extent and mode of cell death of B16 melanoma cells after transfection with mRNA encoding MLKL or tBid. Transfection of B16 cells with tBid-mRNA elicited hallmarks of apoptotic cell death: caspase activity was induced and typical cleavage fragments of caspase-3 were observed, cell death was prevented by the pan-caspase inhibitor zVAD-fmk, and finally cells became sytox positive due to loss of plasma membrane integrity following secondary necrosis (Fig. 1a–c and Supplementary Fig. 2). In contrast, cell death following MLKL-mRNA transfection proceeded without caspase activity or caspase-3 processing, the cells became sytox positive, but in contrast to apoptosis the addition of zVAD-fmk did not prevent cell death, all of which are features of necroptosis (Fig. 1a–c and Supplementary Fig. 2). Cell death induced by mRNA encoding tBid or MLKL did not activate nuclear factor (NF)-κB signaling in the B16 melanoma cells (Fig. 1d). Moreover, the extent of cell death following tBid or MLKL mRNA transfection was comparable in the presence or absence of actinomycin D, suggesting that de novo transcription was not required (Fig. 1e). Time-lapse microscopy confirmed that tBid-mRNA transfection of B16 melanoma cells induced apoptotic membrane blebbing, whereas MLKL-mRNA transfection resulted in cell rounding, followed by swelling and eventually plasma membrane permeabilization (Fig. 1f and supplementary movies 1 and 2)^37^. Finally, to assess the extent of cell death in vivo, B16 melanoma cells were injected subcutaneously (s.c.) into the flank of C57Bl/6 mice, and 6 days later, MLKL- and tBid-mRNA was administered into the tumor (mRNA injection followed by electroporation). Flow cytometric analysis of tumor cells isolated 24 h after in vivo mRNA electroporation revealed that MLKL- and tBid-mRNA electroporation resulted in a very similar level of cell death, which was approximately three-fold higher than the extent of tumor cell death in the saline and irrelevant control mRNA settings (Fig. 1g).

### Intratumor delivery of MLKL-mRNA stalls tumor growth

**Fig. 2:**
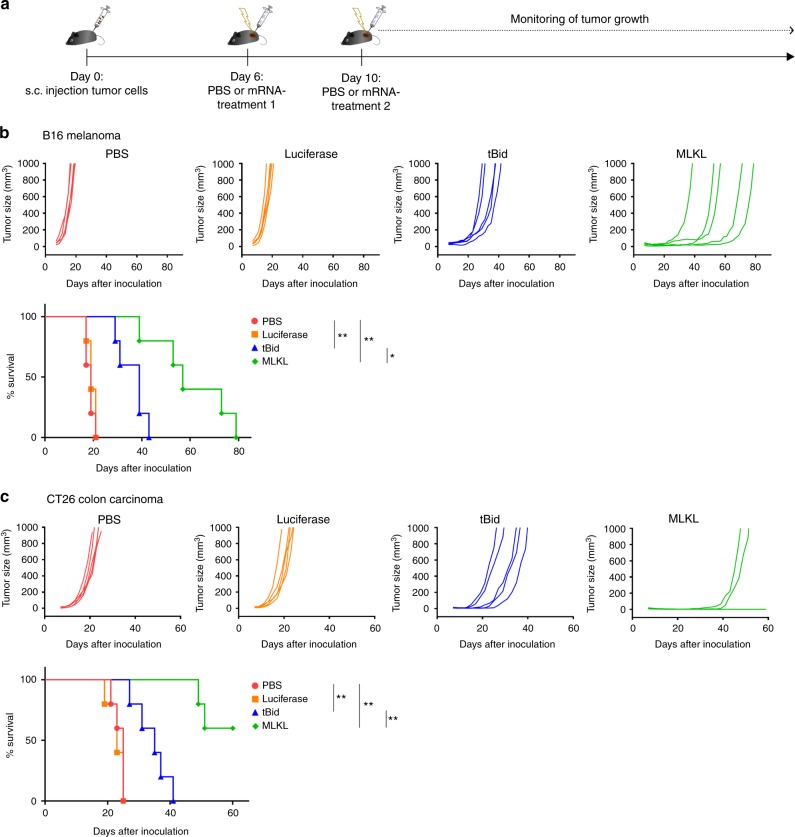
Intratumor MLKL-mRNA protects against primary tumor growth. **a** Schematic representation of the experiment. **b** B16-OVA or **c** CT26-OVA cells were s.c. inoculated in the right flank of C57BL/6J or BALB/cAnNCrl mice (*n* = 5 per group), respectively. On days 6 and 10 (treatment 1 and 2, respectively), the tumors were injected with saline or 10 µg mRNA encoding Fluc, tBid, or MLKL followed by electroporation. Tumor growth was measured over time. The animals were euthanized when the tumor had reached a size of 1000 mm^3^. The results in **b** are representative for three independent experiments. Cox regression analyses showed that there was no difference between the repeats. *p < 0.0.5; **p < 0.01 (log-rank test of Kaplan–Meier curves)

We next evaluated the impact on tumor growth of intratumor delivery of mRNA encoding tBid or MLKL in syngeneic B16 (melanoma)^38^ and CT26 (colon carcinoma)^39^ tumor models. C57BL/6 mice were s.c. inoculated with B16-ovalbumin (B16-OVA) melanoma cells. Established tumors were then treated with saline or mRNAs encoding Fluc (as negative control), tBid, or MLKL according to the schedule shown in Fig. 2a. The tumor growth rate following saline injection and Fluc-mRNA injection followed by electroporation was identical, indicating that mRNA electroporation by itself does not induce adequate immune activation (Fig. 2b and Supplementary Fig. 3). In contrast, intratumor treatment with tBid-mRNA delayed tumor growth and increased the median survival time (time point at which the ethical end point was reached) of the mice from 23 to 43 days (Fig. 2b and Supplementary Fig. 3). However, the most striking antitumor effect was observed for intratumor treatment with MLKL-mRNA: this treatment significantly delayed tumor growth and increased the median survival time to 64 days (Fig. 2b and Supplementary Fig. 3). Since, as noted above, tBid- and MLKL-mRNA treatment induced very similar levels of tumor cell death, we can also conclude that the mere induction of cell death is not sufficient to induce a strong antitumor response.

Genetic and epigenetic alterations in the pathways that lead to necroptosis are common in cancer cells^29^. Although CT26 tumor cells do not express RIPK3^18^, the therapeutic effect of MLKL-mRNA treatment resulted in a very pronounced antitumor effect, increasing the median survival time to >60 days and 60% of the treated mice even remained tumor free up to 80 days after CT26 inoculation (Fig. 2c and Supplementary Fig. 3). Presumably, and in line with a recent report^40^, the level of MLKL expression that is reached after mRNA electroporation of CT26 cells is sufficiently high to elicit cell death in the absence of RIPK3.

**Fig. 3:**
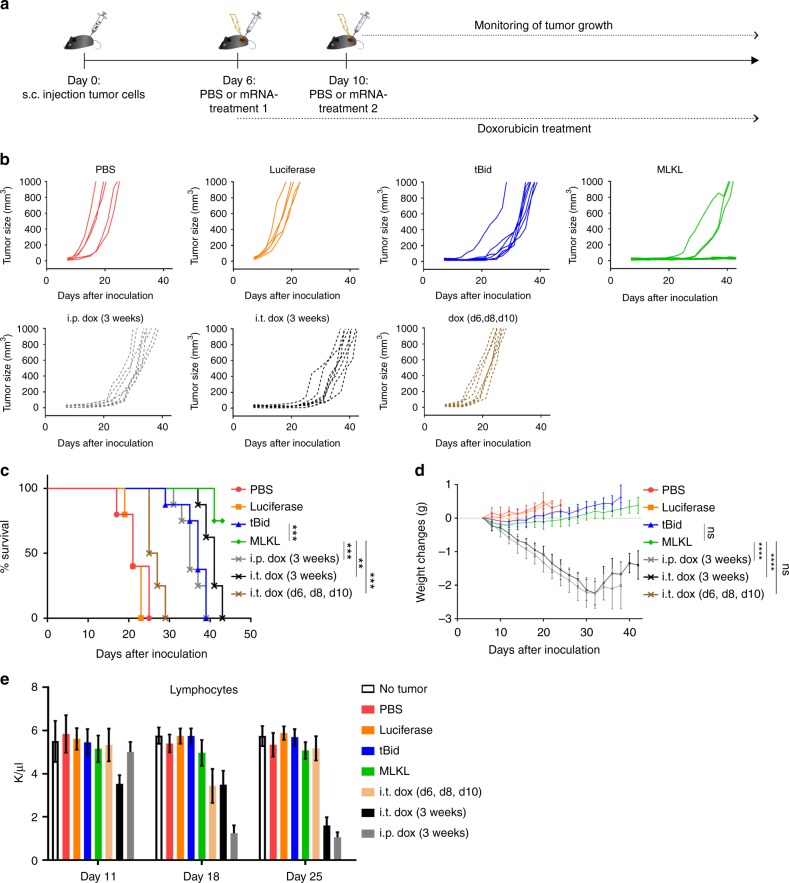
Intratumor MLKL-mRNA protects better than doxorubicin treatment. **a** Schematic representation of the experiment. B16 cells were inoculated s.c. in the right flank of C57BL/6J (*n* = 8 per group). On days 6 and 10, intratumor injection of PBS, Fluc mRNA, tBid mRNA, or MLKL-mRNA followed by electroporation was performed. Doxorubicin (dox, 3 mg/kg per injection) was administered i.p. or intratumorally every second day starting on day 6. One group of mice received 3 intratumor injections of dox on days 6, 8, and 10. **b** Tumor growth progression over time depicted for the individual mice in each group. The animals were euthanized when the tumor had reached a size of 1000 mm^3^. **c** Survival curves and **d** body weight changes of the treated mice. Data points indicate average body weight change relative to day 10 and the error bars depict the standard deviation. **p < 0.01, ***p < 0.001, ****p < 0.0001, ns non-significant determined by log-rank test of Kaplan–Meier survival curves and by one-way ANOVA for the body weight graphs. **e** Hematologic analyses of the number of lymphocytes in blood collected on days 11, 18, and 25 from the treated mice. Each bar represents the average of 8 mice and the error bars depict the standard deviation. The *y* axis depicts the number of lymphocytes per µl of blood

Some of the standard-of-care therapies for cancer patients can induce immunogenic cell death, such as treatment with doxorubicin (dox)^12^. We compared the antitumor effect of the MLKL-mRNA treatment approach with repeated injections of dox in the B16 melanoma model (Fig. 3a). Dox injections were administered every other day into the tumor or intraperitoneally for 3 weeks. Mice from another group were treated on days 6, 8, and 10 only by intratumor injection of dox. MLKL-mRNA treatment was associated with significantly prolonged survival of the mice compared with any of the dox treatment set-ups (Fig. 3b, c and Supplementary Fig. 3). In addition, the prolonged treatment with dox during 3 weeks was associated with significantly reduced body weight compared with the MLKL-mRNA-treated group and with lymphocytopenia (Fig. 3d, e).

### MLKL-mRNA treatment induces systemic antitumor responses

**Fig. 4:**
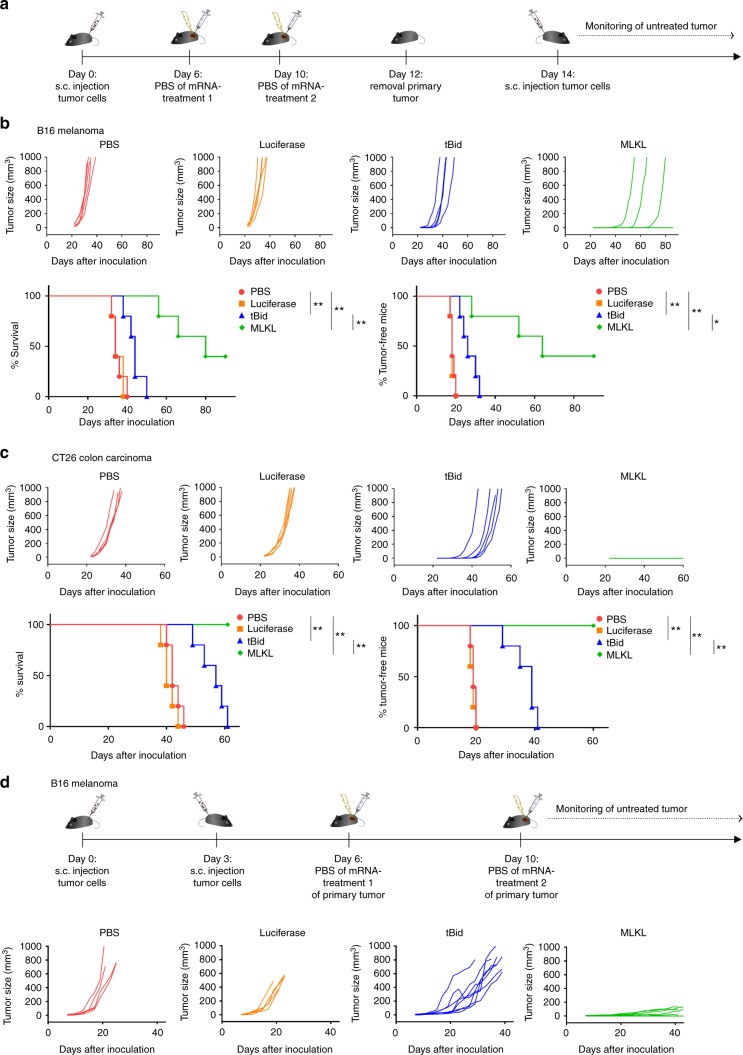
MLKL-mRNA protects against tumor rechallenge and distal tumors. **a** Schematic representation of the treatment and rechallenge experiment. **b** B16-OVA cells or **c** CT26-OVA cells were s.c. inoculated in the right flank of C57BL/6J or BALB/cAnNCrl mice (*n* = 5 per group), respectively. On days 6 and 10 (treatments 1 and 2, respectively), the tumors were injected with saline or 10 µg mRNA encoding Fluc, tBid, or MLKL followed by electroporation. After intratumor treatment, the primary tumor was removed on day 12, and 2 days later, a second inoculation of B16 or CT26 cells in the opposite flank of the mice was performed. Mice were euthanized when tumors reached 1000 mm^3^ in size. The lower graph panels in **b**, **c** depict the percentage of survival (left) and tumor-free mice (right). The results shown in **b** are representative for three independent repeats. Cox regression analyses showed that there was no difference between the repeat experiments. *p < 0.0.5; **p < 0.01 (log-rank test of Kaplan–Meier curves). **d** B16-cells were s.c. inoculated in the right and on day 3 in the left flank of C57BL/6J mice. On days 6 and 10, the tumors on the right flank were injected with saline or 10 µg mRNA encoding Fluc, tBid, or MLKL followed by electroporation. The growth of the untreated tumor that had been inoculated in the left flank was measured over time. Mice were euthanized when the tumor of the right flank had reached a volume of 1000 mm^3^. The experiment was performed once with 5 mice per group in the PBS and luciferase mRNA set-up and 8 mice per group in the tBid- and MLKL-mRNA treatment groups

We hypothesized that the induction of cell death in the tumor by mRNA encoding MLKL could promote the induction of antitumor T cell responses through the release of tumor antigens alongside DAMP^12,41^. Such tumor-specific T cell responses can in principle also obstruct the growth of non-treated distal tumors and metastases. To address whether local intratumor mRNA treatment could induce such an immune-related abscopal effect, we surgically removed the primary tumor 2 days after the second treatment and challenged the mice 2 days later by s.c. injection of B16 or CT26 cells in the opposite flank (Fig. 4a). Intratumor treatment of primary tumors with saline or control mRNA resulted in comparable secondary tumor growth (Fig. 4b, c and Supplementary Fig. 3). tBid-mRNA treatment of the primary tumor provided a modest protection against tumor rechallenge (Fig. 4b, c and Supplementary Fig. 3). However, intratumor treatment of primary tumors with MLKL-mRNA induced a robust protection against tumor rechallenge with 40% of the B16 inoculated mice being tumor free by day 86 and all of the CT26 inoculated mice remaining tumor free up to day 60 of the experiment (Fig. 4b, c and Supplementary Fig. 3).

We also tested the systemic immunity in a second model where a possible abscopal effect could be evaluated^42^. Mice were inoculated with B16 cells in either flank but on different days: the tumor in the left flank was implanted 3 days later than the tumor in the right flank. Only the tumor in the right flank of the animals was subsequently treated, starting at day 6 after the first inoculation. The growth of the distant untreated tumor in the left flank was monitored over time (Fig. 4d). Also, in this set-up, a pronounced delay in tumor growth of the untreated tumor was observed in the case of MLKL-mRNA treatment (Fig. 4d and Supplementary Fig. 3).

**Fig. 5:**
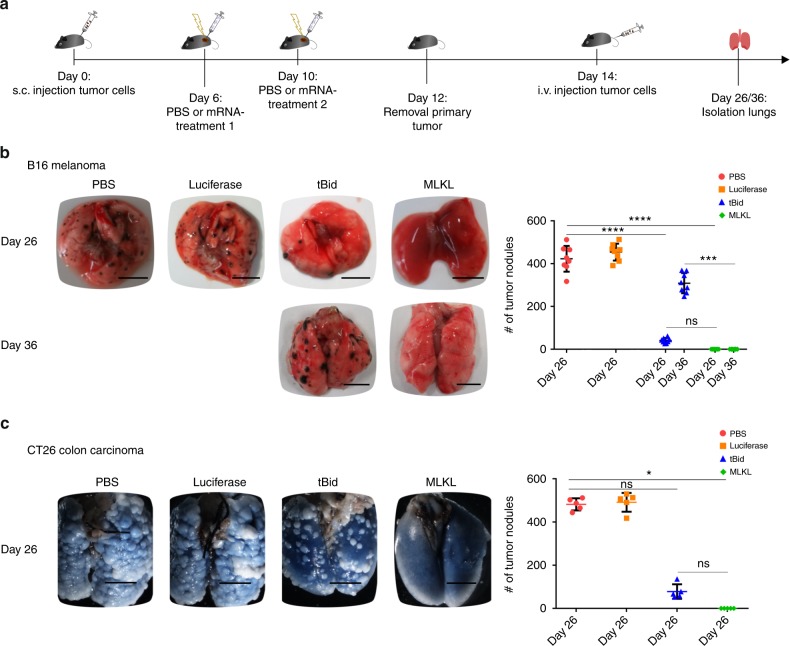
Intratumor MLKL-mRNA protects against experimental lung colonization. **a** Schematic representation of the experiment. B16-OVA (**b**) or CT26-OVA cells (**c**) were s.c. inoculated in the flank of C57BL/6J or BALB/cAnNCrl mice, respectively. After intratumor mRNA treatments 1 and 2 and primary tumor removal, the animals received an intravenous injection of B16-F10 melanoma cells (**b**) or CT26 cells (**c**). Mice were sacrificed 12 or 22 days after i.v. injection, and the number of tumor nodules in the lungs were counted. Results are shown as dot plots, the horizontal lines indicate the mean, and the error bars depict the standard deviation. Results shown in **b** are representative of three independent experiments for the day 26 samples, each with 8 mice per group, and from one experiment for the day 36 sampling with 8 mice per group. The experiment shown in **c** was performed once with five mice per group. *p < 0.0.5; ***p < 0.001; ****p < 0.0001; ns non-significant (Kruskal–Wallis test with Dunn’s post hoc multiple comparison test). In **b**, **c**, the scale bar = 5 mm

Finally, we also evaluated the protective potential of intratumor mRNA delivery in a lung colonization assay. Primary tumors were removed 2 days after the second treatment and mice were subsequently challenged by intravenous (i.v.) injection of B16-F10 or CT26 cells (Fig. 5a). In saline as well as in control mRNA-treated mice, this resulted in the rapid development of tumor nodules in the lungs (Fig. 5b, c). In contrast, intratumor treatment with mRNA coding for MLKL completely protected against lung tumor nodule formation in both the B16 and CT26 models, whereas tBid-mRNA induced only partial protection (Fig. 5b, c).

In summary, intratumor delivery of mRNA encoding MLKL elicits a strong and systemic antitumor immune response that reduces growth not only of the treated tumor but also can prevent secondary local and experimentally disseminated tumor formation.

### MLKL-mRNA combined with anti-PD1 improves antitumor cure

**Fig. 6:**
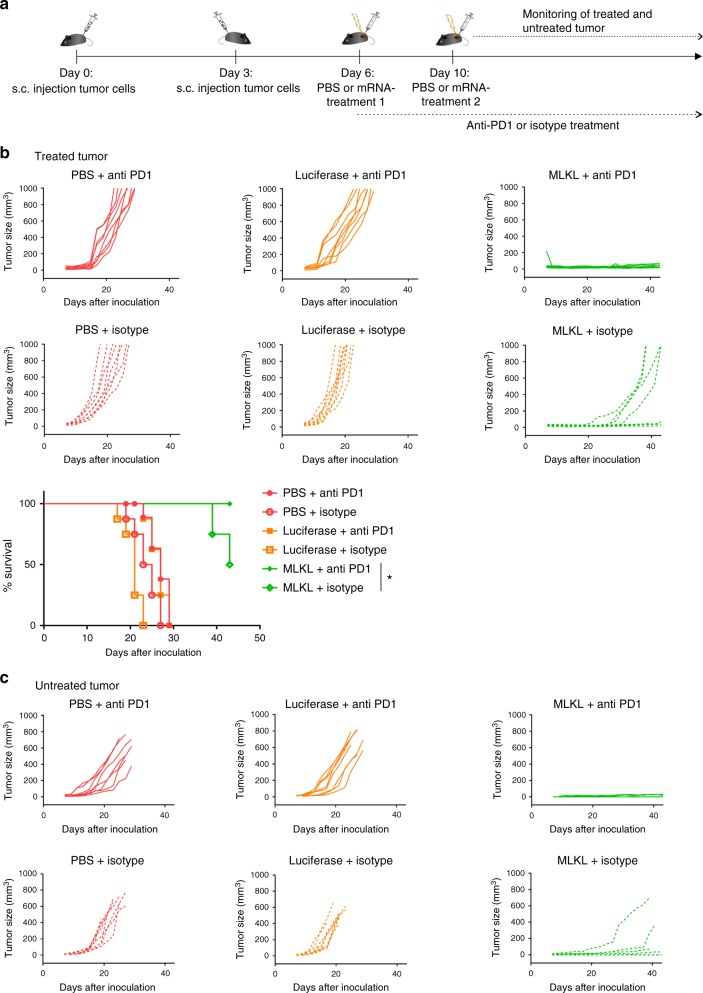
MLKL-mRNA combined with anti-PD1 inhibition improves protection. **a** Schematic representation of the experiment. B16-cells were s.c. inoculated in the right and, on day 3, in the left flank of C57BL/6J mice. On days 6 and 10, the tumor on the right flank was injected with saline or 10 µg mRNA encoding Fluc or MLKL followed by electroporation. Starting from day 6, 200 µg of anti-PD1 or isotype control antibody was administered i.p. every 3 days for 3 weeks or until the ethical end point was reached. The growth of the tumor in the right (**b**) and left flank (**c**) was monitored, and mice were euthanized when the tumor in the right flank had reached a size of 1000 mm^3^. *p < 0.0.5 (log-rank test of Kaplan–Meier curves). The experiment was performed once with eight mice per group

The antitumor activity of MLKL-mRNA treatment might be further improved when combined with cancer treatment options that are already clinically established such as checkpoint blockade approaches. Once inside the tumor bed, T cells primed by intratumor MLKL-mRNA treatment might be silenced by multiple immune-suppressive mechanisms used by tumors to evade elimination^43^. Checkpoint inhibitors such as anti-CTLA4, -PD1, and -PD-L1, indoleamine 2,3-dioxygenase inhibitors or regulatory T depletion strategies primarily act by taking away these breaks. However, these treatments are unfortunately poorly effective in patients with tumors with a low number of tumor-infiltrating T cells^6^. Since the MLKL-based mRNA therapy reported here induces robust infiltration of antigen-presenting cells into the tumor, it is possible that a combination therapy with a checkpoint inhibitor could further improve the curative potential of intratumor delivery of MLKL-mRNA. B16 tumors were implanted s.c. in the right flank of the mice and, 3 days later, in the left flank of the mice. On day 6, the tumor that was inoculated first was treated with MLKL mRNA in combination with i.p. administration of anti-PD1 or an isotype control antibody (Fig. 6a). The anti-PD1 combination therapy was significantly more effective at suppressing the growth of the primary treated tumor and of the distant untreated tumor than the MLKL-mRNA treatment combined with the control antibody (Fig. 6b, c and Supplementary Fig. 3).

### MLKL-mRNA induces protective tumor epitope-specific T cells

**Fig. 7:**
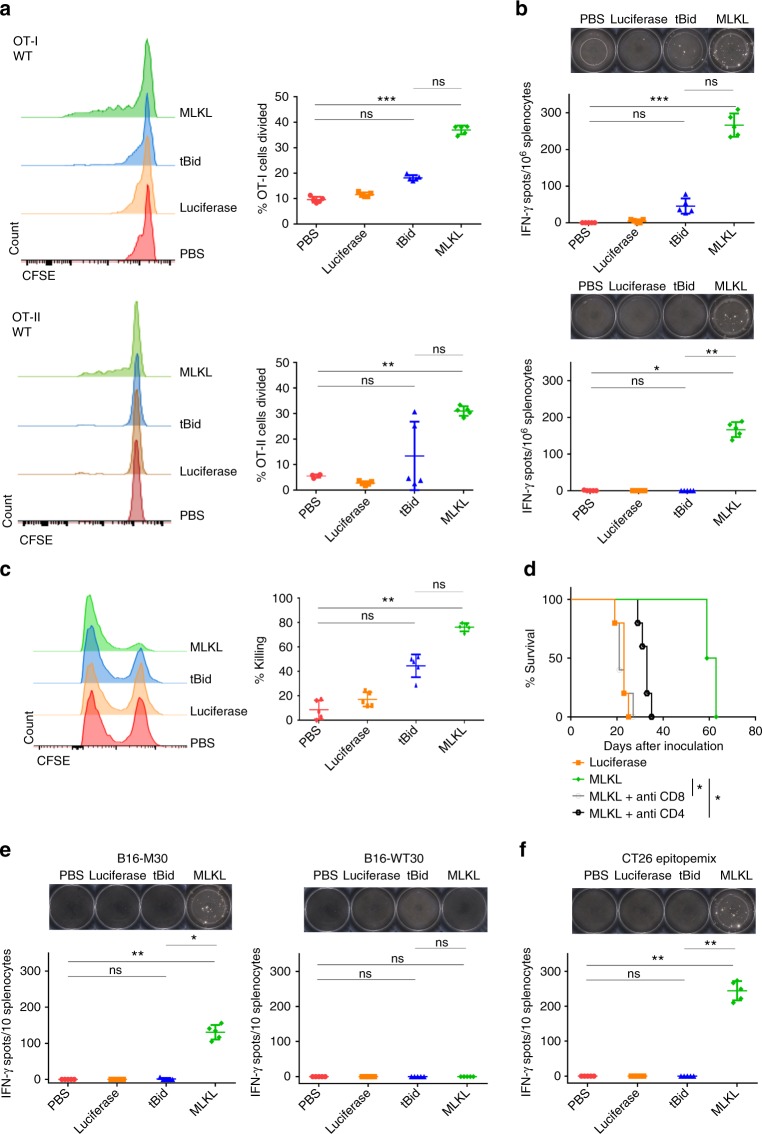
Intratumor treatment with MLKL-mRNA induces antitumor T cell immunity. **a** Flow cytometric analysis of OT-I (top) and OT-II (bottom) proliferation in response to a single intratumor treatment of B16-OVA tumors with saline or 10 µg mRNA encoding Fluc, tBid, or MLKL followed by electroporation (*n* = 5). The results shown are representative for two independent experiments. ***p < 0.001; **p < 0,01; ns = not significant (Kruskal–Wallis test with Dunn’s post hoc multiple comparison test). **b** Induction of OVA MHC I (top) or OVA MHC II (bottom) specific T cell responses after intratumor treatment of B16 tumors analyzed by IFN-γ ELISPOT. Representative ELISPOT pictures are shown above the graphs, which depict the number of spots from individual mice. (*n* = 5). ***p < 0.001; **p < 0.01; *p < 0,05; ns = not significant (Kruskal–Wallis test with Dunn’s post hoc multiple comparison test). **c** Flow cytometric analysis of cytolytic activity as measured by an in vivo killing assay. The results shown are representative for three independent experiments. **p < 0.001; ns = not significant (Kruskal–Wallis test with Dunn’s post hoc multiple comparison test). **d** Kaplan–Meier plot showing the impact of CD4^+^ T cell depletion and CD8^+^ T cell depletion on the antitumor effect of intratumor MLKL-mRNA treatment. *p < 0.05. **e**, **f** Induction of neo-epitope specific T cell responses after intratumor treatment of B16 (**e**) or CT26 (**f**) tumors analyzed by IFN-γ ELISPOT. Representative ELISPOT pictures are shown on top of the graphs, which depict the number of spots from individual mice. In **a**, **b**, **c**, **e**, **f**, horizontal lines indicate the mean and error bars the standard deviation. *p < 0.05; **p < 0.01; ns = not significant (Kruskal–Wallis test with Dunn’s post hoc multiple comparison test)

To address the capacity of intratumor MLKL-mRNA treatment to prime antitumor T cells, we adoptively transferred carboxyfluorescein diacetate succinimidyl ester (CFSE)-labeled OVA-specific transgenic CD8^+^ (OT-I) or CD4^+^ T cells (OT-II) into B16-OVA bearing mice 2 days prior to intratumor mRNA treatment. The tumor draining lymph nodes were excised and T cell proliferation was assessed by flow cytometry 2 days after a single mRNA treatment (Supplementary Fig. 4: gating strategy). We found that only intratumor MLKL-mRNA treatment resulted in significant OT-I and OT-II proliferation compared to control treatment (Fig. 7a). Likewise, only MLKL-mRNA treatment resulted in significant numbers of interferon-γ (IFN-γ) producing OVA-peptide-specific CD4^+^ and CD8^+^ T cells in the tumor draining lymph node (Fig. 7b). The capacity of the primed CD8^+^ T cells to recognize and kill target cells was next evaluated by an in vivo killing assay. Intratumor treatment with MLKL-mRNA was associated with up to 75% specific killing of target cells compared to 40% in mice that had been treated with mRNA encoding tBid, whereas intratumor administration of Fluc-mRNA did not instigate significant target cell lysis (Fig. 7c; Supplementary Fig. 5).

The data above show that the antitumor response induced by intratumor treatment with mRNA encoding MLKL can lead to priming of tumor xeno-antigen-specific CD8^+^ and CD4^+^ T cells and to the elimination of cells that display this antigen. We also found that deletion of CD8^+^ T cells completely and of CD4^+^ T cells partially abolished the antitumor effect evoked by the intratumor treatment with mRNA encoding MLKL (Fig. 7d). These results indicate that CD8^+^ and, to a lesser extent, CD4^+^ T cells contribute for the therapeutic antitumor effect of MLKL-mRNA.

Successful induction of neo-epitope-specific T cell responses is associated with improved therapeutic outcomes in cancer patients. Recently, Kreiter et al. identified several immunogenic neo-epitopes present in B16 and CT26 cells^44^. This enabled us to address whether intratumor treatment with tBid- or MLKL-mRNA could induce T cell responses directed against a subset of these neo-epitopes. In the B16 model, we determined the T cell response directed against the major histocompatibility complex (MHC) class II-restricted neo-epitope B16-M30 or its wild-type counterpart B16-WT30 by IFN-γ-specific ELISPOT. Notably, the peptides corresponding to B16-M30 and -WT30 differ by a single amino acid residue. Splenocytes of B16-bearing mice that had been treated with MLKL-mRNA showed a pronounced reactivity against the mutated B16-M30 neo-epitope but did not respond against the B16-WT30 peptide (Fig. 7e). Neo-epitope-specific T cell responses were also strongly induced in CT26 mice following intratumor MLKL-mRNA administration, and, as in the B16 model, were undetectable after treatment with phosphate-buffered saline (PBS), control or tBid-mRNA (Fig. 7f).

### Migratory Batf3 DCs and type I IFN signaling control antitumor T cells

**Fig. 8:**
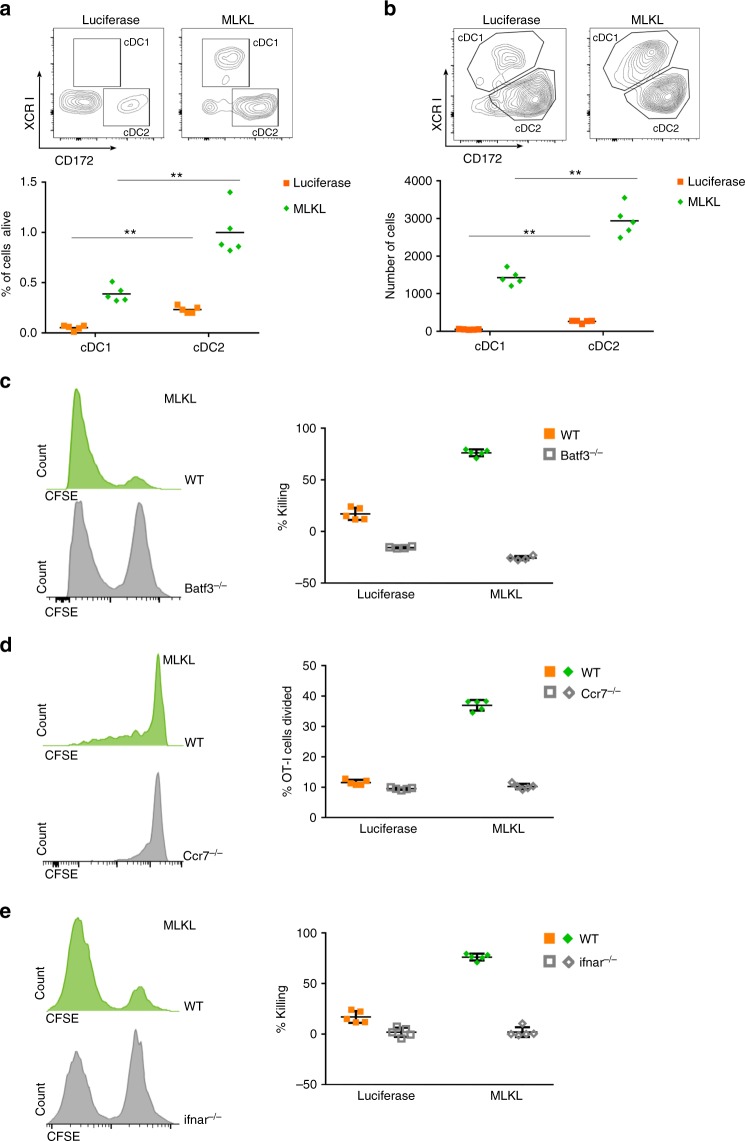
Lymphocyte infiltration and T cell activation depend on Batf3 DCs and type I IFN signaling. C57BL/6J or the indicated knockout mice (*n* = 5 per group) were inoculated with B16-OVA cells and treated once (**a**, **b**) or twice (**c**–**e**) with mRNA encoding Fluc or MLKL. **a** One day after the first treatment, the tumor was dissected and the influx of conventional type 1 (cDC1) and type 2 DCs (cDC2) was analyzed by flow cytometry. **p < 0.01 (Mann–Whitney *U* test). **b** Influx of cDC1 and cDC2 cells in the tumor draining lymph node on day 2 after the first treatment analyzed by flow cytometry. **p < 0.01 (Mann–Whitney *U* test). **c** Three days after the second treatment of B16-OVA tumor carrying wild-type and *Batf3*^−/^^−^ mice, OVA-labeled or irrelevant peptide-pulsed naive CFSE-labeled splenocytes were injected i.v. into the mice and specific killing was determined 2 days later by flow cytometry. **d** Prior to Fluc- or MLKL-mRNA treatment of B16-OVA-bearing wild-type and CCR7^−^^/−^ mice, CFSE-labeled OT-I T cells were adoptively transferred to these mice. On day 14, 4 days after the second treatment, the tumor draining lymph nodes were isolated and OT-I cell proliferation was determined by flow cytometry. **e** Specific killing of OVA-labeled or irrelevant peptide-pulsed naive CFSE-labeled splenocytes injected i.v. 3 days after a second intratumor treatment of B16-OVA-bearing wild-type and *Ifnar1*^−/−^ mice with Fluc-mRNA or MLKL-mRNA. Representative flow cytometric plots or histograms are shown in each panel and the data points are shown as dot plots. In **a**–**e**, horizontal lines indicate the mean. Error bars depict the standard deviation

Type I IFN-mediated activation of the Batf3-dependent CD103^+^ DC subset is critically required for the spontaneous induction of antitumor T cell responses and for the therapeutic benefit of intratumor treatment with Toll-like receptor (TLR) and STING agonists and oncolytic viruses^45–48^. To gain more insight into the immune pathways responsible for the MLKL-mRNA-mediated antitumor responses, we probed the influx of DCs in the tumor bed and in the tumor draining lymph node by flow cytometry on days 1 and 2, respectively, after two intratumor treatments with mRNA encoding MLKL (Fig. 8a, b; Supplementary Fig. 6). Batf3-dependent DCs (conventional type 1 DCs or cDC1) and IRF4-dependent DCs (cDC2) represent the two major classes of DCs and can be discriminated by their distinct expression of XCR1 (cDC1) versus CD172α (cDC2). Compared to mock treated mice, a strong influx of cDC1 and cDC2 DCs was apparent in the tumor bed and its draining lymph nodes in mice that had been treated intratumorally with mRNA coding for MLKL (Fig. 8a, b). To address the role of Batf3 DCs in the induction of CD8^+^ T cell responses, we compared the cytolytic activity between wild-type mice and *Batf3-*deficient mice^49^. In contrast to wild-type mice, B16-OVA-inoculated *Batf3* knockout mice did not mount an OVA-specific cytotoxic T cell response upon intratumor treatment with MLKL-mRNA, confirming the crucial involvement of the CD8α DC subset for T cell priming (Fig. 8c). Furthermore, initial priming of CD8^+^ T cells depended on the migration of DCs to the draining lymph nodes, since mice that are deficient in CCR7, the chemokine receptor crucial for the migration of tissue DCs to lymph nodes, displayed defective OT-I T cell proliferation upon intratumor treatment with MLKL-mRNA of B16-OVA tumors (Fig. 8d). Finally, we assessed the involvement of type I IFN signaling in the therapeutic benefit of intratumor MLKL-mRNA treatment with an in vivo cytolytic activity assay in *Ifnar1*^−/−^ mice. IFNAR1 deficiency totally abrogated the cytolytic activity in response to intratumor MLKL-mRNA treatment, demonstrating the necessity of type I IFN signaling (Fig. 8e).

### MLKL-mRNA stalls tumor growth in humanized mice

**Fig. 9:**
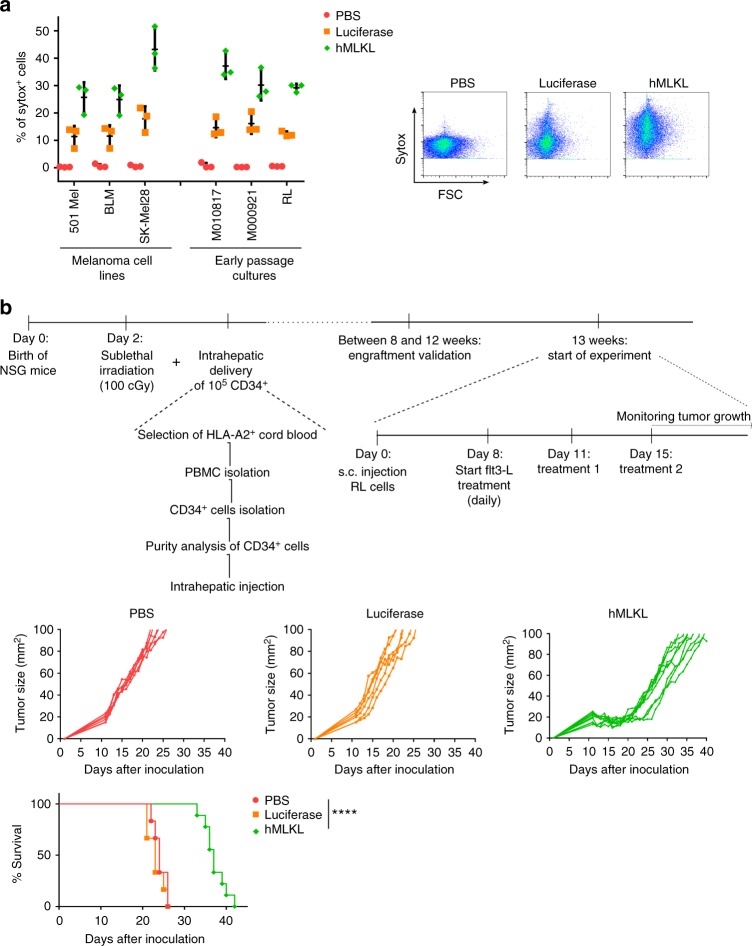
Intratumor MLKL-mRNA treatment reduces tumor growth in humanized mice. **a** Human melanoma cell lines (501 Mel, BLM, SK-Mel28), human early passage cultures (M010817 and M000921), and human B lymphoma cells (RL cells) were transfected with PBS or with mRNA encoding Fluc or human MLKL. Twenty four hours after transfection, cells were collected and analyzed by flow cytometry. The graph shows the percentages of sytox^+^ cells (left) and flow cytometric plots of transfected RL cells (right). The horizontal lines indicates the mean and the error bars depict the standard deviation. **b** Newborn NSG mice (2 days) were sublethally irradiated and subsequently received 1 × 10^5^ CD34^+^ human stem cells isolated from HLA-A2-positive cord blood by injection in the liver. Thirteen weeks after stem cell transfer, 2.5 × 10^6^ human RL follicular lymphoma cells were inoculated s.c. into the mice. On days 11 and 15 (treatments 1 and 2, respectively), the tumors were injected with saline (*n* = 6) or 10 µg mRNA encoding Fluc (*n* = 6) or human MLKL (*n* = 9) followed by electroporation. Starting 8 days after tumor inoculation and during the treatments, 30 µg Flt3 ligand was given daily. Tumor growth was measured over time. The animals were culled when the tumor had reached a size of 100 mm^2^. ****p < 0.001 (log-rank test of Kaplan–Meier curves)

In a final set of experiments, we exploited the potential of mRNA coding for human MLKL (hMLKL) to induce an antitumor effect. We first evaluated the sensitivity of different human melanoma cell lines and early passage human tumor cells to cell death induced after transfection of mRNA encoding hMLKL. Flow cytometric analyses of mRNA-transfected human melanoma cell lines, early passage melanoma cells, and RL human B lymphoma cells showed that, unlike mock transfection, Fluc-mRNA and more extensively hMLKL-mRNA transfection resulted in cell death (Fig. 9a).

To assess the therapeutic potential of intratumor hMLKL-mRNA treatment of a human tumor in vivo, we used mice with a humanized adaptive immune system. Irradiated newborn NOD-SCID-gamma (NSG) mice that had received an intrahepatic injection of human CD34^+^ stem cells, were inoculated s.c. with 2.5 × 10^6^ human RL follicular lymphoma cells. At days 11 and 15, when a palpable tumor could be observed, the tumors were treated with saline or with mRNA encoding Fluc or hMLKL (Fig. 9b). Intratumor administration of saline or mRNA encoding Fluc resulted in comparable tumor growth. However, a striking antitumor effect following intratumor treatment with hMLKL-mRNA was observed, which significantly delayed tumor growth and increased the median survival time of the mice (Fig. 9b). In a repeat experiment in this human tumor model, MLKL mRNA outperformed tBid mRNA treatment in controlling tumor growth (Supplementary Fig. 9 and Supplementary Table 1). These results suggest that an hMLKL-mRNA-based antitumor treatment could be beneficial in a clinical setting.

## Discussion

**Fig. 10:**
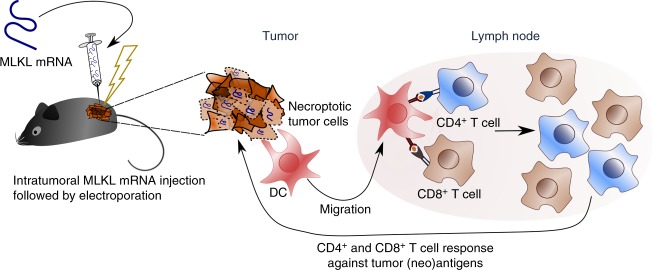
Proposed mechanism of intratumor MLKL-mRNA treatment. In vitro transcribed mRNA encoding MLKL is injected in an established tumor and the tumor is subsequently electroporated. In this way, the MLKL-mRNA can enter cells in the tumor mass and its evokes cell death. Batf3 DCs are attracted to the dying tumor cells and will take up tumor-specific antigen and subsequently crosspresent it in the draining lymph node to CD4^+^ and CD8^+^ T cells. These primed CD4^+^ and CD8^+^ T cells can then mount a cellular response directed against tumor-specific (neo)antigens

Stimuli that turn the immune-suppressive TME into a milieu that is favorable for the activation of antitumor T cell responses can induce potent antitumor immunity. In this study, we describe an approach to elicit protective antitumor immunity based on the intratumor administration of hypo-inflammatory mRNA that encodes the necroptotic cell death executioner protein MLKL. This approach elicited tumor antigen-specific CD4^+^ and CD8^+^ T cell responses that stalled primary tumor growth and protected against a rechallenge with the parental tumor cells. Strikingly, MLKL-mRNA treatment of the tumors elicited strong T cell responses directed against tumor neo-epitopes (Fig. 10). Mechanistically, we found that Batf3-dependent DCs and type I IFN signaling were vital to raise tumor-specific T cell responses upon MLKL-mRNA treatment. It has been reported that chemically induced RIPK3 dimerization can trigger immunogenic cell death and antitumor immunity by a process that requires NF-κB-dependent cytokine expression and the production of inflammatory cytokines by the dying tumor cells^17^. However, we found no evidence that NF-κB was activated following MLKL mRNA transfection. To address the question whether an intratumor MLKL-mRNA treatment method holds promise for clinical application, we performed experiments in mice with a grafted human immune system that were subsequently inoculated with HLA-matched human lymphoma-derived cancer cells. In these mice, hMLKL-mRNA treatment strongly suppressed tumor growth, suggesting that this approach could be effective in the clinic.

Necroptosis can be induced by death receptor ligation, toll-like receptor stimulation, or intracellular microbial nucleic acid sensing, which precipitates on the activation of RIPK3 that in turn phosphorylates MLKL. The phosphorylation of MLKL results in its oligomerization and recruitment to the cell membrane where the protein is thought to induce plasma membrane destabilization by mechanisms that are still unclear. Some models favor direct pore formation^23,24^ and others mention the requirement for the recruitment of ion channels^25^. Recently, the ESCRT III mechanism has been implicated in the regulation of the formation of MLKL-containing exosomes^50–52^. Prophylactic immunization of mice with cancer cells that are dying by necroptosis induced by conditional activation of a RIPK3 transgene construct can induce antitumor immunity^17,18^. Nonetheless, in situ induction of necroptosis in tumors represents a major challenge, as many tumor types display genetic and epigenetic alterations in the pathways leading to necroptosis. For this reason, we opted to use mRNA encoding a downstream executioner of necroptosis, i.e., MLKL. This strategy enabled us to induce necroptotic cell death and subsequent antitumor immunity even in the context of the RIPK3-deficient CT26 tumor model. Therefore, loss of RIPK3 and MLKL expression as observed in many cancer cells would likely not compromise the MLKL-mRNA approach described here. The efficiency of the antitumor effect of the MLKL-mRNA treatment correlates with the extent of necroptosis-like cell death that is induced in the tumor cells and not with the extent of induced cell death per se, since tBid-mRNA treatment, which induced a comparable degree of cell death as MLKL-mRNA, resulted in very poor antitumor activity. It is surprising that transfected mRNA encoding wild-type MLKL could kill cells because it has been reported that induced expression of wild-type MLKL in mouse dermal fibroblasts failed to do so^22^. We speculate that the MLKL expression levels in mRNA-transfected cells rapidly reach a critical concentration that could lead to the formation of amyloid-like polymers that induce necroptosis^40^.

Intratumor MLKL-mRNA treatment induced T cell responses against several neo-epitopes with known protective potential^44^. It has also been reported that the induction of T cell responses directed against mutated tumor neo-epitopes is associated with improved clinical outcome. As T cells that recognize these mutated neo-epitopes have not been subjected to central tolerance induction, they are considered to be of superior quality as compared to those recognizing non-mutated antigens^53,54^. Based on these findings, active immunization strategies are being pursued to evoke T cells recognizing an individual patient’s tumor neo-epitopes^55^. A limitation of such a strategy is the critical dependency on the reliability of the algorithms used to predict the immunogenicity of mutated peptide sequences. In addition, neo-epitopes are highly specific for a given tumor and patient, which implies that for each patient a personalized new vector or delivery system has to be generated to elicit neo-epitope-specific responses^56,57^. The MLKL-mRNA-based therapy described here resulted in the clear induction of tumor antigen-specific CD4^+^ and CD8^+^ T cell responses without a necessity for tumor sequencing, epitope prediction, and production of a personalized vaccine vector (Fig. 6e, f). Therefore, we speculate that our approach could be developed as an intervention to induce tumor antigen-specific T cell responses early on in cancer patients. This could trigger a protective adaptive immune response, buy time for subsequent characterization of the tumor mutanome by exome sequencing and bio-informatic prediction tools, and subsequently boost the primed immune response induced by the MLKL-mRNA treatment with, for example, a follow-up therapeutic vaccination based on the identified neo-epitopes.

Mechanistically, we provide evidence that the antitumor T cell responses induced by MLKL-mRNA treatment require Batf3-dependent cDC1, DC migration, and type I IFN signaling (Fig. 7c–e). Hence, MLKL-mRNA treatment may deploy the same cells and signaling cascades that have been described to be vital for spontaneous antitumor T cell immunity, for T cell-related abscopal effects of radiotherapy^58^, and for the therapeutic benefit of intratumor injection of TLR agonists^59^, STING agonists^60^, and oncolytic viruses^61,62^.

The successful therapeutic outcome in mice with a humanized immune system upon hMLKL-mRNA-based anticancer treatment described here is an important step toward clinical application. Manufacturing and clinical translation of mRNA-based drugs has been established in recent years by several companies^55,63,64^. Identifying clinically implementable ways of delivering MLKL-mRNA into tumors is an important hurdle to overcome on the path from bench side to the clinic. Injection of mRNA followed by electroporation as described in this study might be primarily applicable to easily accessible tumors, such as melanoma or head and neck tumors. It is important to note that an increasing armory of delivery vehicles is being developed by academic groups and companies to deliver mRNA directly to tumors in vivo^65^, opening avenues to also treat visceral tumors with MLKL-mRNA.

## Methods

### Cell lines and culture conditions

Cells were cultured in Dulbecco’s modified Eagle’s medium supplemented with 10% of fetal calf serum, 2 mM of L-glutamine, 0.4 mM of Na-pyruvate, non-essential amino acids, 100 U/ml of penicillin, and 0.1 mg/ml of streptomycin at 37 °C in a humidified atmosphere containing 5% CO_2_. Murine tumor cells used were melanoma cell lines (B16, B16-OVA, B16-F10) and colon carcinoma cells (CT-26). Human melanoma cell lines (501 Mel, BLM, SK-mel28) and early passage cultures (M018017 and M000921) were kindly provided by Dr. Geert Berx from Ghent University. RL and B16 cells were purchased from the American Type Culture Collection (ATCC) and cultured in conditions specified by the manufacturer. CT26 cells were provided by the laboratory of Professor Patrizia Agostinis (KU-Leuven). No full authentication was performed on the cell lines and all cell lines tested negative for mycoplasma contamination.

### Production of in vitro transcribed mRNA

The coding information for Fluc, mouse tBid, mouse MLKL, and human MLKL were cloned into the in-house generated plasmid vector pIVTstab that contains a T7 promoter, 5’ and 3′ untranslated region of human β globulin, and a poly A_60_ tail. The mRNA expression plasmids pIVTstab-GFP, pIVTstab-Luc, pIVTstab-tBid, and pIVTstab-MLKL were propagated in *Escherichia coli* MC1061 cells (Stratagene, La Jolla, CA, USA) and purified using endotoxin-free QIAGEN-tip500 columns (Qiagen, Chatsworth, CA, USA). The MLKL and tBid encoding plasmids were linearized with *Pst*I (New England Biolabs, MA, USA), whereas the OVA, GFP, and Fluc encoding plasmids were linearized with *Spe*I (New England Biolabs, MA, USA). The linearized plasmids were purified using a PCR purification kit (Roche, Upper Bavaria, Germany). The mRNA was produced with the T7 mMessage Machine Kit (Ambion, Austin, Tx, USA) according to the manufacturer’s instruction. 5-Methylcytidine and pseudo-uridine (TriLink, San Diego, CA, USA) were used in the transcription reactions instead of cytidine and uridine, respectively. The in vitro transcribed mRNA was purified by lithium chloride precipitation and subsequently simultaneously capped and 2′-O-methylated to synthesize Cap 1 RNA from uncapped RNA using the ScriptCap m^7^G Capping System Kit together with the ScriptCap 2′-O-methyltransferase Kit (Ambion, Austin, TX, USA) according to the manufacturer’s instruction. The capped in vitro transcribed mRNA was purified by lithium chloride precipitation.

### In vitro transfection

Cells were plated 24 h before transfection in a 6- or 96-well plate at 10^6^ or 10^4^ cells/well, respectively. One million B16 cells were transfected with 1 µg of mRNA complexed with Lipofectamine® RNAiMAX (Life Technologies, Ghent; Belgium) diluted in OptiMem to obtain a total volume of 300 µl. The transfection mix was added to the cells and cells were incubated at 37 °C, 5% of CO_2_ during a time period depending on the experiment. Transfection efficiency was evaluated by measuring uptake of Cy-5-labeled eGFP mRNA and the onset of translation of the transfected mRNA by determining GFP fluorescence at different time points after transfection using a triple-laser (B-V-R) LSR-II (Becton Dickinson, San Jose, CA, USA) flow cytometer. The flow cytometric data were analyzed with FlowJo (Treestar, OR).

### Analysis of cell death and caspase activity

B16 cells (10^6^ cells/well in 6-well plate) were analyzed at different time points after transfection with saline or 1 µg of mRNA encoding Fluc, tBid, or MLKL. The extent of membrane permeability was assessed by staining with Sytox Blue Nucleic Acid Stain (4000X dilution) (Molecular Probes, S11348). The cells were analyzed on a triple-laser (B-V-R) LSR-II (Becton Dickinson, San Jose, CA, USA). First single cells were selected based on their forward scatter (FSC) and side scatter (SSC). Next dead cells were identified based on SYTOX blue positivity (Supplementary Fig. 2: gating strategy). The flow cytometric data were analyzed with FlowJo (Treestar, OR).

To analyze caspase activity and cell death, a FLUOstar OMEGA (BM, Labtech) assay was performed. Therefore, 5 × 10^3^ cells were seeded in a transparent 96-well plate and transfected with saline or 5 ng of mRNA encoding Fluc, tBid, or MLKL. In all, 2 µM of SYTOX Green nucleic acid stain (Molecular Probes (S7020) and 33 mM of Ac-DEVD-AMC (Peptanova, 317-V) was added to the cells. Cell death was measured based on SYTOX Green fluorescence (excitation 485 nm, emission 520 nm) and set relative to the signal of 0.05% of Triton X-100-treated cells. Caspase activity was measured by cleavage of Av-DEVD-AMC into fluorescent 7-amino-4-methylcoumarin (AMC) (excitation 355 nm, emission 460 nm). The DEVDase activity is expressed as fold induction compared to the maximal fluorescence intensity value of cells treated with 10,000 U/ml tumor necrosis factor (TNF; eBioscience) and 2 µM TAK inhibitor (Analyticon Discovery GmbH).

To analyze MLKL protein expression and caspase 3 cleavage, a western blot was performed. B16 cells (10^6^ cells/well in 6-well plate) were transfected with saline or 1 µg of mRNA encoding Fluc, tBid, or MLKL. Twenty four hours after transfection, the lysates were separated by sodium dodecyl sulfate-polyacrylamide gel electrophoresis (10% acrylamide) and MLKL and caspase-3 were visualized by western blotting with, respectively, anti-MLKL (1000× dilution) (Millipore, Cat. No. MABC604) and anti-caspase 3 (1000× dilution) (Cell Signaling Technology, Cat. No. 9662S) antibodies.

To analyze the possible induction of NF-κB upon cell death evoked by the transfected mRNAs, a luciferase assay was performed. B16 cells were seeded at 5 × 10^4^ cells per well in 24-well plates 24 h before transfection. Cells were transfected with 50 ng of a plasmid in which the coding sequence of luciferase is under the control of the NF-κB promoter (pConluc) and 100 ng of a plasmid expressing β-galactosidase (pB-act-B-galactosidase). Twenty four hours later, the B16 cells were transfected with saline or 100 ng of mRNA encoding GFP, tBid, or MLKL, or as a positive control, the B16 cells were transfected with 25 ng TRAF6 expression plasmid (pcDNA3.1-HA-TRAF6) or stimulated with 100 U/ml TNF. At different time points, cells were lysed with luciferase lysis buffer (25 mM Tris-phosphate, 2 mM dithiothreitol, 2 mM CDTA, 10% glycerol, and 1% Triton X-100). Luciferase activity was measured with a GloMax® 96 Microplate Luminometer (Promega) by adding luciferin to the lysates. To normalize the luciferase activity, the B-galactosidase activity was measured on a iMark microplate reader (Biorad). The ratio of the β-galactosidase and luciferase activities was determined to normalize for transfection efficiency.

To investigate whether the induced cell death requires transcription, a flow cytometric experiment was performed. B16 cells (10^6^ cells/well in 6-well plate) were transfected with 1 µg of the GFP-expressing plasmid pMAX-GFP combined with saline or 1 µg of mRNA encoding Fluc, tBid, or MLKL. Half of the transfected cells were treated with actinomycin D (1 µg/ml) immediately after transfection. Twenty four hours after transfection, the extent of membrane permeability was assessed by staining with Sytox Blue Nucleic Acid Stain (4000× dilution) (Molecular Probes, S11348). The cells were analyzed on a triple-laser (B-V-R) LSR-II (Becton Dickinson, San Jose, CA, USA). First single cells were selected based on their FSC and SSC. Next dead cells and GFP-expressing cells were identified based on SYTOX blue positivity (Supplementary Fig. 2: gating strategy) or GFP positivity. The flow cytometric data were analyzed with FlowJo (Treestar Inc., USA).

### Live cell imaging

B16 cells were seeded at 15 × 10^3^ cells per well in an 8-well chamber (iBidi) in 200 µl of complete growth medium. Twenty four hours later, cells were transfected with saline or 1 µg of mRNA encoding Fluc, tBid, or MLKL just before imaging. Live cell imaging was performed on a Leica Sp5 AOBS confocal microscope (Leica), using an ×40 HCX PL Apo UV 1.25 na oil objective. Images were acquired in a sequential mode every 30 min.

### Mice

Female C57BL/6 mice were purchased from Charles River France and female BALB/cAnNCrl mice from Charles River Italy (via France). OT-I mice carrying a transgenic CD8^+^ T cell receptor specific for the MHC-I-restricted OVA peptide (257-264), OT-II mice carrying a transgenic CD4^+^ T cell receptor specific for the MHC-II-restricted OVA peptide (323–339), *CCR-7*-, *batf3-*, and *IFNAR1-*deficient mice were bred at the breeding facility of the VIB-UGent Center for Inflammation Research (VIB, Ghent, Belgium). NSG mice were bred at the breeding facility of the University Hospital Ghent (UZ Ghent, Belgium). All mice were aged 7–10 weeks at the start of the experiment. Animals were housed under specific pathogen-free conditions in individually ventilated cages in a controlled 12-h day–night cycle and given food and water ad libitum. All procedures involving animals were approved by the local Ghent University ethics committee (accreditation nr. LA1400536, Belgium), in accordance with European guidelines. Mice experiments are covered under the following EC applications: EC2016-010 and ECD17/11. The mice were allocated randomly to a group. The investigators were not blinded during data collection or analysis.

### In vivo bioluminescence imaging

For in vivo imaging, mice were inoculated s.c. with 5 × 10^5^ B16 cells. Six days after inoculation 10 µg of mRNA coding for Fluc was injected into the tumor. To monitor Fluc activity, 150 mg/kg of D-luciferin (PerkinElmer, Waltham, MA, USA) in PBS was injected i.v. at different time points after Fluc mRNA injection. The mice were sedated and monitored using an IVIS lumina II imaging system. Photon flux was quantified using the Living Image 4.4 software (all from Caliper Life Sciences, Hopkinton, MA, USA).

### In vivo mRNA electroporation

C57BL/6 mice and BALB/cAnNCrl mice that had been inoculated s.c. with tumor cells were shaved at the site of tumor growth. A total of 10 µg of mRNA dissolved in 10 µl of Hank’s Balanced Salt Solution (HBSS; Gibco) was injected into the tumor using a U-100 insulin needle (BD Biosciences, San Diego, CA, USA). Next a conductive gel (EKO-GEL, ultrasound transmission gel, Egna, Italy) was applied at the tumor site to ensure electrical contact of the electrodes with the skin and electroporation was performed. Two pulses of 20 ms and 120 V/cm were delivered through spaced plate electrodes by an ECM® 830 Electroporation System (BTX ® Harvard apparatus). Mice were treated this way on days 6 and 10 after tumor cell inoculation.

### Tumor implantation and tumor growth measurement

In total, 5 × 10^5^ B16 (OVA) cells or CT26 cells suspended in 100 μl of HBSS were injected s.c. into the right flank of each C57BL/6 or BALB/cAnNCrl mice, respectively. At days 6 and 10 after inoculation of the tumor cells, mRNA was injected into the tumor and the tumor was subsequently electroporated. For the comparison of the mRNA treatment with an antracyline treatment, B16 inoculated mice received dox (3 mg/kg) at days 6, 8, and 10 or during 3 weeks every 2 days. Unless otherwise indicated, these dox doses were injected perilesionally, s.c. next to the tumor border. For the combination therapy, 200 µg of anti-PD1 from BioXcell (Cat. No. BE0146) or an isotype control antibody from BioXcell (Cat. No. BE0089) was injected intraperitoneally during 3 weeks every 3 days, starting on day 6 after inoculation of the first tumor. Depending on the set-up of the experiment, the primary tumor was removed and 5 × 10^5^ B16 or CT26 cells diluted in 100 μl of HBSS were injected s.c. into the left flank of each C57BL/6 or BALB/cAnNCrl mice, respectively, or 2 × 10^5^ B16F10 or CT26 cells were injected i.v. The tumor size was measured every 2 days with an electronic digital caliper. The tumor volume was calculated as the length × width × height (in mm^3^). The mice were humanely euthanized when the volume of the tumor reached 1000 mm^3^. For the experimental lung colonization assay experiments, mice were euthanized 12 days after i.v. injection of the tumor cells and tumor nodules were counted. In the CT26 model, lung tumor burden was quantified after tracheal ink (1:10 diluted in PBS) injection and fixation with Fekete’s solution (5 ml 70% ethanol, 0.5 ml formalin, and 0.25 ml glacial acetic acid).

### Analysis of DC infiltration in the tumor draining lymph nodes

B16 cells (5 × 10^5^) diluted in 100 μl of HBSS were injected s.c. into the right flank of each C57BL/6 mouse. At day 6 and 10 after inoculation of the tumor cells, mRNA was injected into the tumor and the tumor was subsequently electroporated. Two days after the second treatment, the draining lymph nodes were dissected and passed through 70 µm nylon strainers (BD Biosciences, San Diego, CA, USA) to obtain single-cell suspensions. Cells were stained with anti-CD16/CD32 (500× dilution) (BD Biosciences) to block Fc receptors followed by staining with Fixable Viability Dye efluor 780 (1000× dilution) (eBioscience), Ly6C-FITC (400× dilution) (BD Biosciences), XCR1-BV510 (300× dilution) (Biolegend), CD172α-PerCP-efluor710 (200× dilution) (eBioscience), CD64-biotin BV786 SA (300× dilution) (Biolegend), CD207-AF647 (200× dilution) (eBioscience), CD11c-PE-efluor610 (800× dilution) (eBioscience), MHCII-AF700 (800× dilution) (eBioscience), CD3-PE-Cy5 (200× dilution) (eBioscience), and CD19-PE-Cy5 (400× dilution) (eBioscience). Cell analysis was performed on a five-laser fortessa (Becton Dickinson, San Jose, CA, USA), and data were analyzed using the FlowJo software (Treestar, OR). First, live single cells were identified based on SSC, FSC, and live/dead stain. T and B cells were gated out based on their CD3 and CD19 positivity, respectively. Subsequently, monocyte-derived DCs were identified as CD64^+^MHCII^+^ cells, cDC1 as CD64^−^ MHCII^+^ CD11c^+^ XCR1^+^, and cDC2 as CD64^−^ MHCII^+^ CD11c^+^ CD127α^+^ cells. See Supplementary Fig. 6 for gating strategy.

### In vivo T cell proliferation assay

Two days prior to mRNA immunization, 2 × 10^6^ OT-I or OT-II cells were purified and labeled with 5 µM of CFSE (Invitrogen, Merelbeke, Belgium) and subsequently adoptively transferred via i.v. injection into mice that had been s.c. inoculated with B16 cells. Four days after the mRNA treatment, draining lymph nodes were isolated and OT-I or OT-II cell division was analyzed by flow cytometry. Cells were stained with anti-CD16/CD32 (500× dilution) (BD Biosciences) to block Fc receptors followed by staining with Fixable Viability Dye (1000× dilution) (BD Biosciences), CD8 PE-Cy7 (200× dilution) (eBiosciences), CD3 efluor450 (200× dilution) (eBioscience), anti-CD19 allophycocyanin (APC; 200× dilution) (BD Biosciences), and MHC-I dextramer H-2 Kb/SINFEKL-PE (500× dilution) (Immundex). The experiments were performed on a triple-laser (B-V-R) LSR-II (Becton Dickinson, San Jose, CA, USA), and data were analyzed using the FlowJo software (Treestar, OR). Single cells were gated based on FSC and SSC. Living cells were selected and T cells gated for CD3^+^ CD19^−^ T cells. Within the CD8^+^ T cells or CD4^+^ T cells, OVA-specificity was gated by labeling with MHC-I SINFEKL-PE dextramer. See Supplementary Fig. 4 for the gating strategy.

### In vivo killing assay

Splenocytes from female mice were pulsed with 1 µg/ml of the MHC-I-restricted OVA_257-264_ peptide or HIV-1 gag peptide as a control before labeling with 5 or 0.5 µM of CFSE (Invitrogen, Merelbeke, Belgium), respectively. Labeled cells were mixed at a 1:1 ratio and a total of 1.5 × 10^7^ mixed cells were adoptively transferred into immunized mice 3 days after the second mRNA treatment. Forty eight hours later, splenocytes from mice were isolated and passed through 70 µm nylon strainers (BD Biosciences, San Diego, CA, USA) to obtain single-cell suspensions. Red blood cells were lysed using ACK red blood cell lysis buffer (BioWhittaker, Wakersville, MD, USA). Next, splenocytes were analyzed by flow cytometry. Percentage of antigen-specific killing was determined using the following formula: (1 − (%CFSE^hi^ cells/%CFSE^low^ cells)^treated mice^/(%CFSE^hi^ cells/% CSFE^low^ cells)^non-treated mice^) × 100. The experiments were performed on a triple-laser (B-V-R) LSR-II (Becton Dickinson, San Jose, CA, USA) and analyzed using the FlowJo software (Treestar, OR). Single cells were gated based on their SSC and FSC. Next CFSE-positive cells were selected. See Supplementary Fig. 5 for gating strategy.

### ELISPOT analysis

C57BL/6 mice were inoculated with 5 × 10^5^ B16 cells, and at days 6 and 10, the mice were treated with saline or 10 µg mRNA encoding Fluc, tBid, or MLKL. Two days after the second treatment, spleens were isolated and passed through 70 µm nylon strainers (BD Biosciences, San Diego, CA, USA) to obtain single-cell suspensions. Red blood cells were lysed using ACK red blood cell lysis buffer (BioWhittaker, Wakersville, MD, USA) and 2.5 × 10^5^ cells were cultured for 24 h in wells of anti-IFN-γ (Diaclone, Besancon, France) pre-coated 96-well plates in the presence of 10 µg/ml peptide. The following synthetic, high-performance liquid chromatography-purified peptides were used for restimulation: OVA 257–264 (SIINFEKL), OVA 323–339 (SQAVHAAHAEINEAGR), CT26-M20 (PLLPFYPPDEALEIGLELNSSALPPTE), CT26-M26 (VILPQAPSGPSYATYLQPAQAQMLTPP), CT26-M03 (DKPLRRNNSYTSYIMAICGMPLDSFRA), CT26-M37 (EVIQTSKYYMRDVIAIESAWLLELAPH), CT26-M27 (EHIHRAGGLFVADAIQVGFGRIGKHFW), B16-M30 mut (PSKPSFQEFVDWENVSPELNSTDQPFL), and B16-M30 WT (PSKPSFQEFVDWEKVSPELNSTDQPFL).

### CD8^+^ and CD4^+^ depletion experiments

In total, 5 × 10^5^ B16 cells diluted in 100 μl of HBSS were injected s.c. into the right flank of each C57BL/6 mouse. At days 6 and 10 after inoculation of the tumor cells, mRNA was injected into the tumor and the tumor was subsequently electroporated. In the CD8^+^ depletion assay, 200 µg of anti-mouse CD8α antibody (clone YTS 169.4, BioXCell) was i.p. injected on days 5 and 10 after tumor inoculation. In the CD4^+^ depletion assay, 200 µg of anti-mouse CD4 antibody (clone GK1.5, BioXCell) was i.p. injected at days 3, 6, and 9 after tumor inoculation. The tumor size was measured every 2 days with an electronic digital caliper as described above.

### Mice with a humanized immune system

To obtain hematopoietic stem cells (HSC) that HLA type matched with the human RL tumor cells used for the antitumor experiment, cord blood cells were stained with HLA-A2-FITC (BD Pharmingen) or with HLA-ABC-PE (BD Pharmingen) as a positive control prior to HSC isolation. Samples were acquired on an Attune Nxt Acoustic Focusing Cytometer (Life Technologies). HLA.A2^+^ samples were selected for CD34^+^ stem cells. To that end, viable mononuclear cells were isolated by gradient separation to isolate the viable mononuclear cells. Next, CD34^+^ cells were isolated using a direct CD34^+^ progenitor cell isolation kit (Miltenyi). To evaluate the purity of the isolated stem cells, a flow cytometric staining was performed (human CD3-PE (BD Pharmingen)/human-CD34-APC (BD Pharmingen)). Samples were acquired on an Attune Nxt Acoustic Focusing Cytometer (Life Technologies). The purity of the injected cells reached 92–98%. To generate mice with a humanized immune system, newborn NSG mice (2 days old) received a sublethal irradiation of 100 cGy followed by an intrahepatic delivery of CD34^+^ stem cells. Eight and 12 weeks after CD34^+^ stem cell transfer, peripheral blood was analyzed for the presence of both human and mouse CD45^+^ cells to analyze engraftment (Supplementary Fig. 7 and 8). Samples were acquired on an LSR flow cytometer (BD) and analyzed by FACS Diva software (BD). The mice were s.c. inoculated with 2.5 × 10^6^ human RL follicular lymphoma cells at 13 weeks after stem cell transfer. From day 8 onwards, the mice received a daily intraperitoneal injection of 30 µg of recombinant human FLT3-L protein. On days 11 and 15 after the RL cell injection, the tumors were injected with saline or with 10 µg of mRNA encoding Fluc, human tBid, or MLKL followed by electroporation. Tumor growth was measured over time. The animals were euthanized when the tumor had reached a size of 100 mm^2^.

### Hematologic analysis

On days 11, 18, and 25, blood was collected from the tail vein in EDTA-coated microvette tubes (Sarstedt) and analyzed in a Hemavet 950FS (Drew Scientific) whole blood counter.

### Statistical analyses and data presentation

Statistical analyses were performed using the GraphPad Prism 7 software. Cox regression analyses showed that there was no difference between the three repeats of Figs. 2b and 3b. Statistical significance between survival rates was done by comparing Kaplan–Meier curves using log-rank test. Statistical significance between experimental groups was assessed using Kruskal–Wallis test (with Dunn’s post hoc multiple comparison test, as stated). Non-parametric or group comparisons with small sample sizes were assessed by unpaired two-tailed Mann–Whitney *U* test.

### Data availability

All data supporting the findings of this study are available within the article or from the corresponding author upon request.

## Electronic supplementary material

Supplementary Information

Peer Review

Description of Additional Supplementary Files

Supplementary Movie 1

Supplementary Movie 2
