# Supplementary material for: Treatment with mRNA coding for the necroptosis mediator MLKL induces antitumor immunity directed against neo-epitopes
Source: Nat Commun. 2018 Aug 24;9:3417. doi: 10.1038/s41467-018-05979-8 (PMC6109072; doi:10.1038/s41467-018-05979-8)
Supplement: Supplementary file 1 — Supplementary Information [file 41467_2018_5979_MOESM1_ESM.pdf]

Supplementary Information:

Treatment with mRNA coding for the necroptosis mediator MLKL induces antitumor immunity directed against neo-epitopes

Lien Van Hoecke[1,2], Sandra Van Lint[1,3], Kenny Roose[1,2], Alexander Van Parys[1,3], Peter Vandenabeele[2,4], Johan Grooten[2], Jan Tavernier[1,3], Stefaan De Koker[2], Xavier Saelens[1,2,*]

[1]VIB-UGent Center for Medical Biotechnology, VIB, Ghent, Belgium

[2]Department of Biomedical Molecular Biology, Ghent University, Ghent, Belgium

[3]Cytokine Receptor Laboratory, Department of Biochemistry, Faculty of Medicine and Health Sciences, Ghent University, Ghent, Belgium

[4]VIB-UGent Center for Inflammation Research, VIB, Ghent, Belgium

*To whom correspondence should be addressed: xavier.saelens@vib-ugent.be

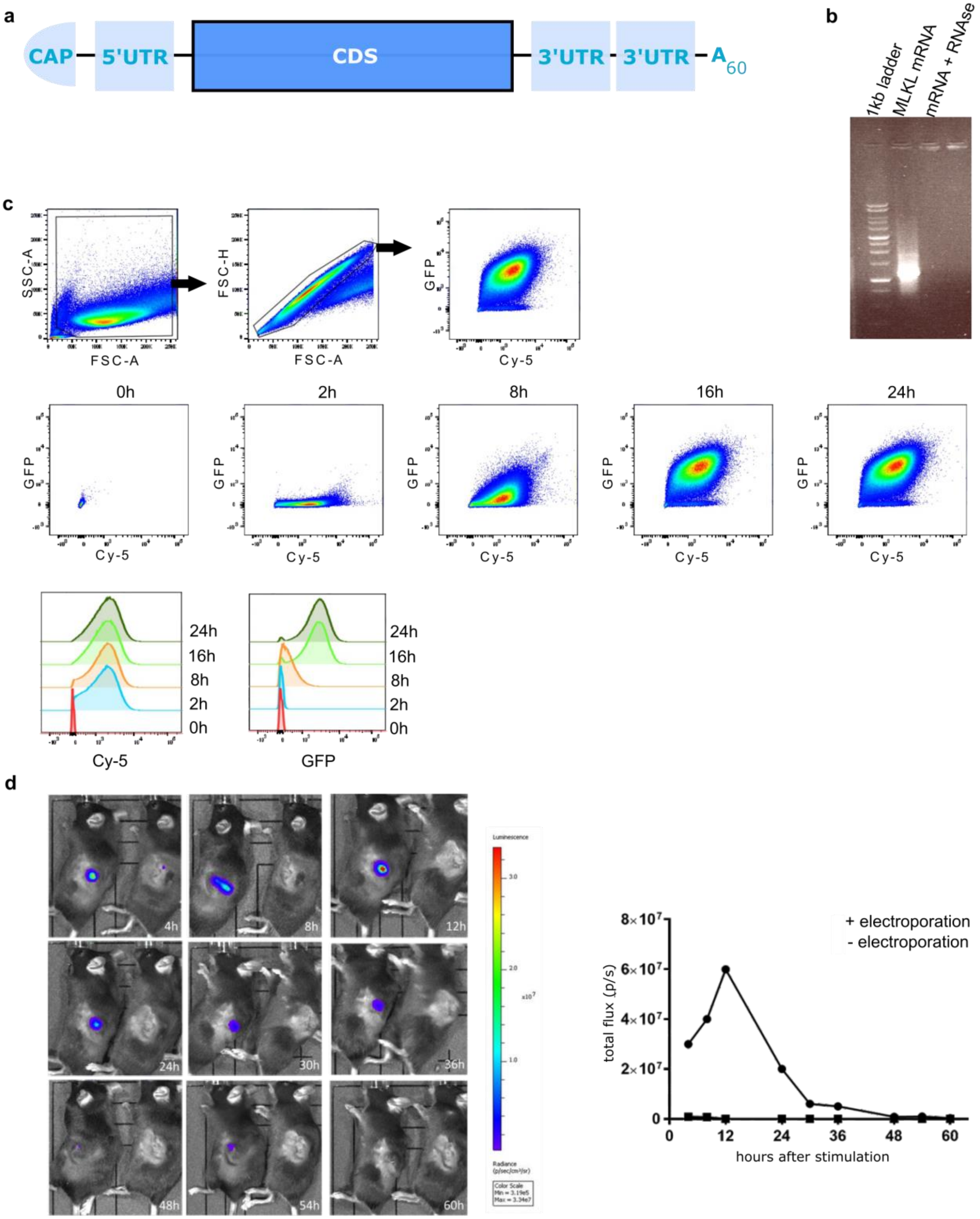

a
CAP
5'UTR
CDS
3'UTR
3'UTR
A60
b
1kb ladder
MLKL mRNA
mRNA + RNAse
c
SSC-A
FSC-A
FSC-H
FSC-A
GFP
Cy-5
0h
2h
8h
16h
24h
GFP
Cy-5
24h
16h
8h
2h
0h
Cy-5
GFP
d
4h
8h
12h
24h
30h
36h
48h
54h
60h
+ electroporation
- electroporation
total flux (p/s)
hours after stimulation

Supplementary Figure 1: *In vitro* and *in vivo* characterization of designed mRNAs. (a) Designed hypo-inflammatory mRNAs. The 5’ and tandem duplicated 3’ untranslated region (UTR) of the human β-globulin (HBB) was added upstream and downstream of the coding sequences to increase the stability of the mRNAs. A 60 nucleotide poly-A tail was added 3’ of the constructs. An O-methylated 5’ m7-cap was ligated postranscriptionally to the *in vitro* produced mRNAs. (b) Purified *in vitro* transcribed mRNA coding for MLKL with or without RNAse A treatment was loaded on a 1% agarose gel. (c) *In vitro* mRNA transfection and translation efficiency. Cy5-labeled mRNA coding for GFP was transfected into B16-OVA cells. At different time points after transfection Cy5 and GFP fluorescence were analyzed by flow cytometry. Cy5 positivity is a measurement of transfection efficiency while GFP positivity is a measurement of translation efficiency. The upper panels depict the gating strategy. The middle and bottom panels show Cy5 and GFP fluorescence determined at different time points (0, 2, 8, 16 and 24h) after mRNA transfection. (d) *In vivo* reporter gene expression efficiency. C57BL/6J mice were subcutaneously inoculated with 5E5 B16-OVA cells in the flank. Six days later, the mice were injected in the tumor with 10 µg Fluc-encoding mRNA followed by electroporation (left mouse) or not (right mouse). D-luciferine was injected intraperitoneally at different time points after mRNA injection and the Fluc activity was measured by whole body imaging.

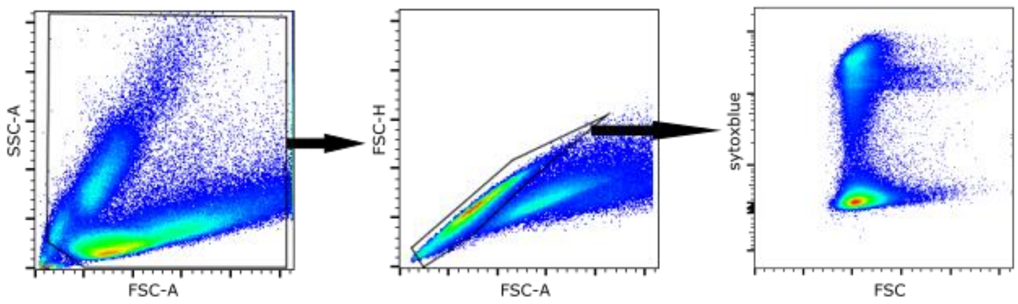

Supplementary Figure 2: gating strategy for Sytox blue staining. First single cells were selected based on the FSC and SSC. Next sytox blue positivity was analyzed.

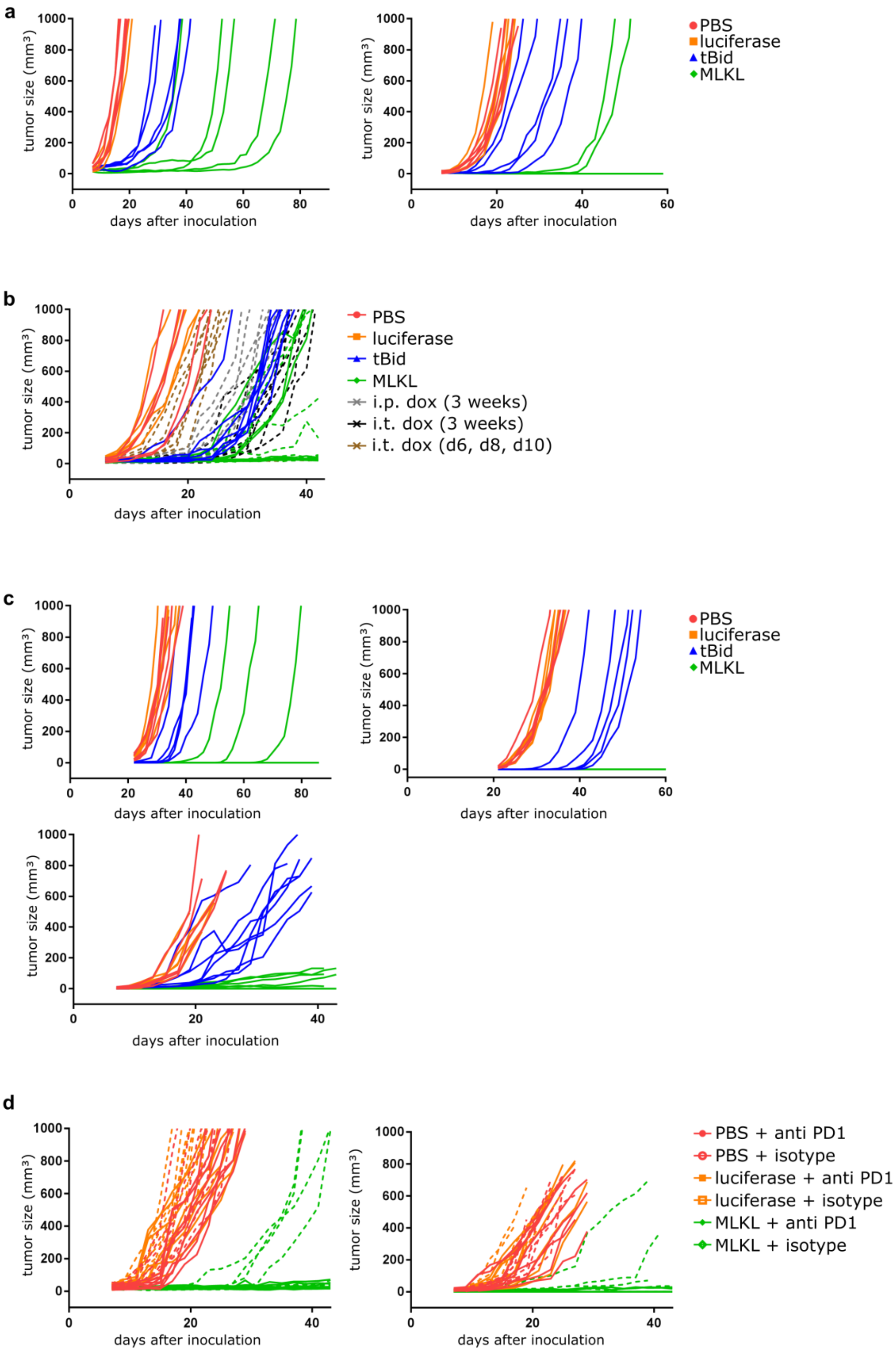
a
b
c
d
tumor size (mm³)
days after inoculation
PBS
luciferase
tBid
MLKL
i.p. dox (3 weeks)
i.t. dox (3 weeks)
i.t. dox (d6, d8, d10)
PBS + anti PD1
PBS + isotype
luciferase + anti PD1
luciferase + isotype
MLKL + anti PD1
MLKL + isotype

Supplementary Figure 3: Overlay of tumor growth curves. a: Overlay of the tumor growth curves corresponding to Figure 2b (left) and Figure 2c (right). b: Overlay of the tumor growth curves corresponding to Figure 3b. c: Overlay of the tumor growth curves corresponding to Figure 4b (left), Figure 4c (middle) and Figure 4d (right). d: Overlay of the tumor growth curves corresponding to Figure 6b (left) and 6c (right).

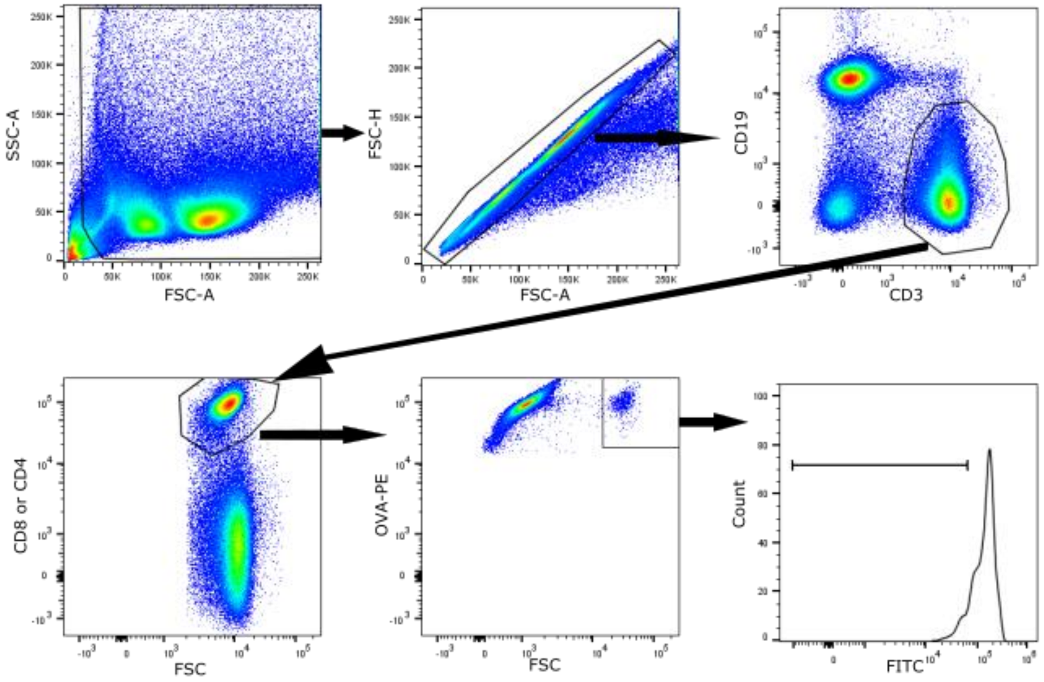

Supplementary Figure 4: gating strategy for OT-I or OT-II proliferation. First single cells were selected based on the FSC and SSC. Next, T cells were gated as $CD3^+$ $CD19^-$ cells. In this T cell population $CD8^+$ T cells (in OT-I proliferation assay) or $CD4^+$ T cells (in OT-II proliferation assay) were selected. The FITC profile of the OVA^+ $CD8^+$ T or $CD4^+$ T cells was analyzed.

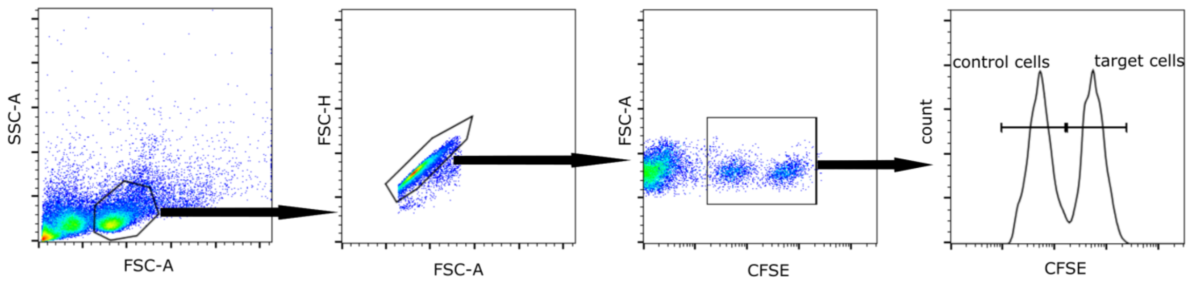

Supplementary Figure 5: gating strategy for the *in vivo* killing assay. First single cells were selected based on the FSC and SSC. Next the CFSE profile was analyzed in the CFSE^{+} population.

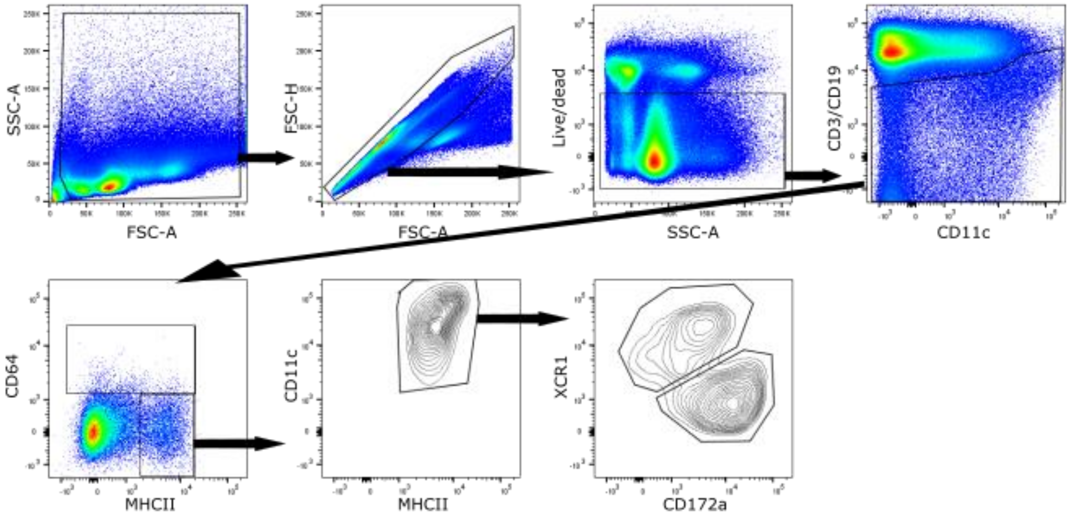

Supplementary Figure 6: gating strategy dendritic cells. First living single cells were selected based on the FSC, SSC and live/death staining. Next, T cells and B cells were gated out based on the CD3 and CD19 expression, respectively. cDC1 cells were identified as $MHCII^{+}CD11c^{+}XCR1^{+}CD172\alpha^{-}$ cells and cDC2 cells were identified as $MHCII^{+}CD11c^{+}CD172\alpha^{+}$ $XCR1^{-}$ cells.

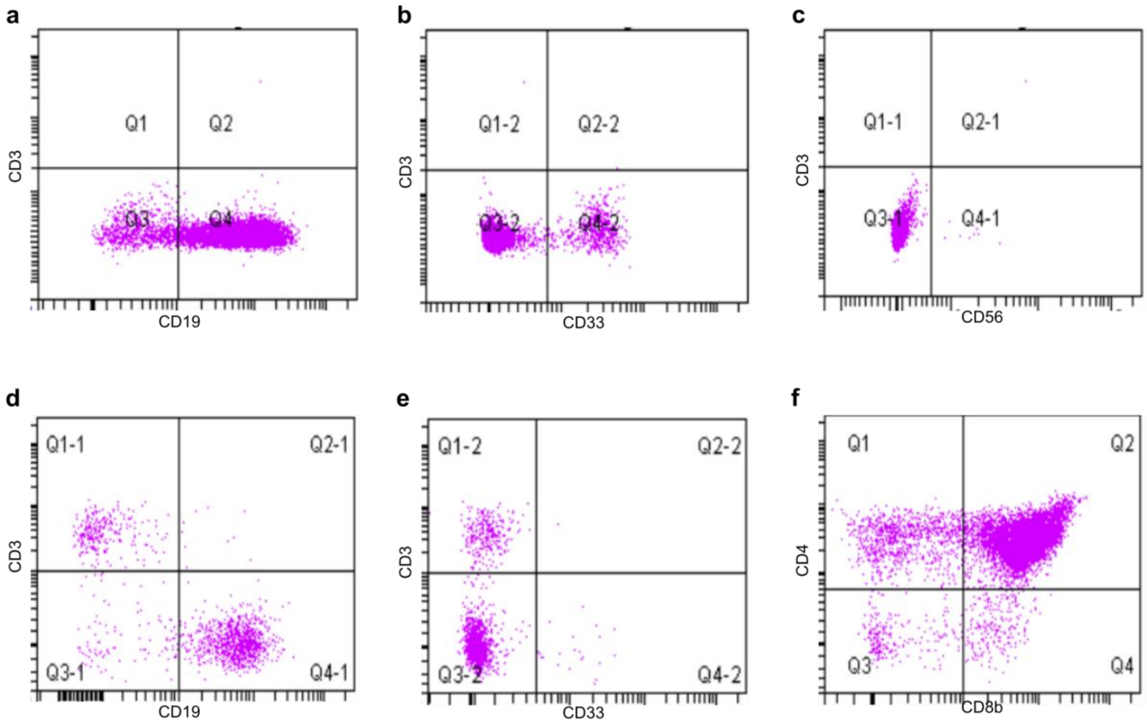

Supplementary Figure 7: Representative analysis of human immune system reconstitution in organs of irradiated and engrafted NSG mice. Bone marrow cells from the left and right femur and tibia were isolated, blocked with human and mouse Fc-block and stained with live/dead fixable aqua, anti-human CD45, anti-mouse CD45, and anti-human CD3, CD19, CD33 and CD56 as indicated. Human immune cell reconstitution was analysed on viable hCD45$^+$ cells using flow cytometry. Large populations of human B cells (hCD19$^+$; panel a) and human myeloid cells (hCD33$^+$; panel b) could be observed. In addition, a small population of Natural Killer cells (hCD56$^+$; panel c) was present. The spleen and thymus were isolated and grinded to obtain single cell suspensions. Spleen cells were blocked with human and mouse Fc-block and stained with live/dead fixable aqua, anti-human CD45, anti-mouse CD45, and anti-human CD3, CD19, and CD33 as indicated. When gated on viable hCD45$^+$ cells, distinct populations of human B and T cells were present (panel d), while only few myeloid cells were detected (panel

e) in the spleen of HIS mice. The thymus cell suspension was blocked with human and mouse Fc-block and stained with live/dead fixable aqua, anti-human CD45, anti-mouse CD45, and anti-human CD3, CD4 and CD8b as indicated. When gated on viable $hCD45^+CD3^+$ T cells, we found that immature $CD4^+CD8^+$ double positive T cells as well as mature $CD4^+$ and $CD8^+$ single positive T cells were present (panel f), showing that human T cells undergo maturation and differentiation in the thymus of the grafted mice.

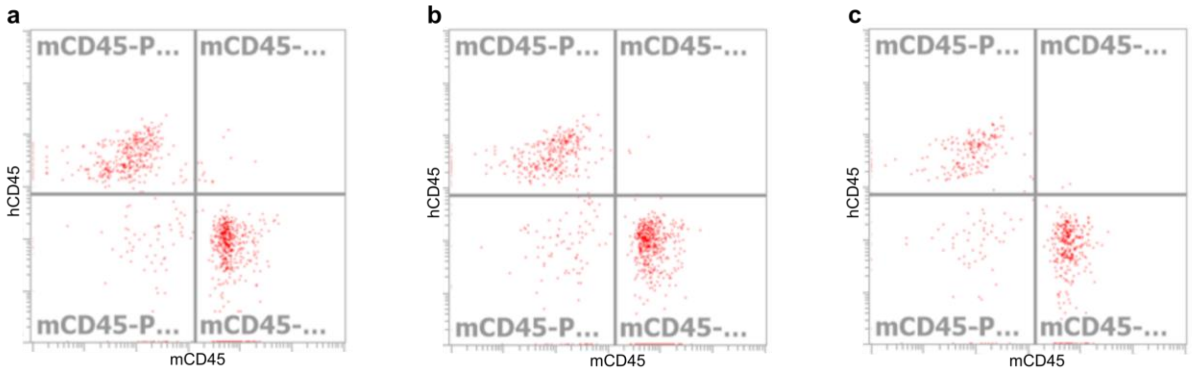

Supplementary Figure 8: Peripheral blood analysis of mice with a reconstituted human immune system. Eight weeks after intrahepatic injection of human haematopoietic stem cells, human cell engraftment in the peripheral blood was evaluated. Ten microliter peripheral blood per mouse was collected from the tail vein and treated with lysis buffer to remove red blood cells. Cell suspensions were subsequently blocked with human and mouse Fc-block and stained with live/dead fixable aqua, anti-human CD45 and anti-mouse CD45 (human and mouse pan-leukocyte markers, respectively). Viable blood cells were analysed by flow cytometry for the presence of a distinct $hCD45^+$ population. Panels a-c represent the results for human cell engraftment in the peripheral blood of 3 different reconstituted mice.

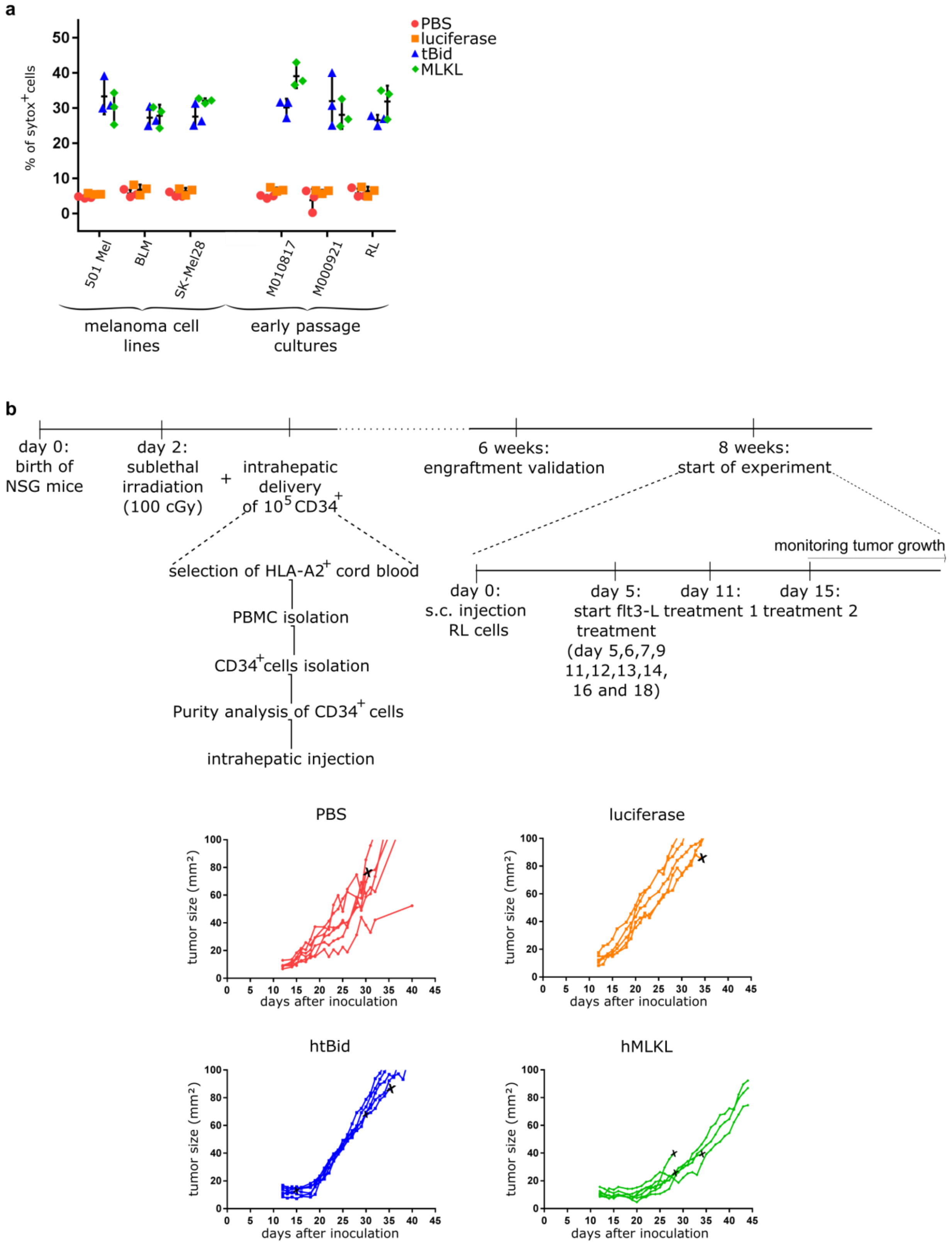
a
PBS
luciferase
tBid
MLKL
% of sytox+cells
501 Mel
BLM
SK-Mel28
M010817
M000921
RL
melanoma cell lines
early passage cultures
b
day 0: birth of NSG mice
day 2: sublethal irradiation (100 cGy)
+
intrahepatic delivery of 10^5 CD34+
6 weeks: engraftment validation
8 weeks: start of experiment
selection of HLA-A2+ cord blood
PBMC isolation
CD34+cells isolation
Purity analysis of CD34+ cells
intrahepatic injection
monitoring tumor growth
day 0: s.c. injection RL cells
day 5: start flt3-L treatment (day 5,6,7,9 11,12,13,14, 16 and 18)
day 11: treatment 1
day 15: treatment 2
PBS
luciferase
htBid
hMLKL
tumor size (mm²)
days after inoculation

Supplementary Figure 9: Intratumor MLKL-mRNA treatment protects better against primary tumor growth of human RL cells than tBid-mRNA in mice with a humanizes immune system. (a) Human melanoma cell lines (501 Mel, BLM, SK-Mel28), human early passage cultures (M010817 and M000921) and human B lymphoma cells (RL cells) were transfected with PBS or with mRNA encoding Fluc, human tBid or human MLKL. Twenty four hours after transfection the cells were collected and analyzed by flow cytometry. The graph shows the percentages of sytox+ cells. (b) Newborn NSG mice (2 days) were sublethally irradiated and subsequently received $1x10^5$ $CD34^+$ human stem cells isolated from HLA-$A2^+$ positive cord blood by injection in the liver. Eight weeks after the stem cell transfer, 2,5 x 10^6 human RL follicular lymphoma cells were inoculated s.c. into the mice. On days eleven and fifteen (treatment 1 and 2, respectively) the tumors were injected with PBS (n = 6) or 10 µg mRNA encoding Fluc (n = 5), human tBid (n = 6) or human MLKL (n = 6) followed by electroporation. At day five, six, nine, eleven, twelve, thirteen, fourteen, sixteen and eighteen, 30 µg recombinant human Flt3 ligand was administered to the mice by intraperitoneal injection. Tumor growth was measured over time. The animals were euthanised when the tumor had reached a size of 100 mm^2. One mouse in the PBS and luciferase mRNA and 3 mice in the tBid and MLKL mRNA groups died during the experiment before the tumor size had reached the ethical endpoint.

Supplementary Table 1. Human immune cell engraftment in reconstituted NSG mice. The table gives an overview of the allocation of the humanized mice to the PBS, irrelevant, tBid and MLKL mRNA treatment groups shown in Supplementary figure S9. Only mice with an engraftment of at least 5% $hCD45^{+}$ cells of viable peripheral blood cells were used in the experiment.

Experimental group	hCD45+ (%)	Experimental group	hCD45+ (%)
**PBS**	20,1	**htBid**	9.4
**PBS**	5,8	**htBid**	10
**PBS**	6	**htBid**	10.7
**PBS**	9,7	**htBid**	7.3
**PBS**	6,9	**htBid**	7.6
**PBS**	7,2	**htBid**	14
**IRR mRNA**	6.9	**hMLKL**	6.2
**IRR mRNA**	5.9	**hMLKL**	6
**IRR mRNA**	11.9	**hMLKL**	9
**IRR mRNA**	5.4	**hMLKL**	61.9
**IRR mRNA**	15	**hMLKL**	8.1
		**hMLKL**	18.4
