# Supplementary material for: Treatment with mRNA coding for the necroptosis mediator MLKL induces antitumor immunity directed against neo-epitopes
Source: Nat Commun. 2018 Aug 24;9:3417. doi: 10.1038/s41467-018-05979-8 (PMC6109072; doi:10.1038/s41467-018-05979-8)
Supplement: Supplementary file 3 — Description of Additional Supplementary Files [file 41467_2018_5979_MOESM3_ESM.pdf]

Description of Additional Supplementary Files

File Name: Supplementary Movie 1
Description: Time lapse of B16 cells transfected with mRNA encoding MLKL.

File Name: Supplementary Movie 2
Description: Time lapse of B16 cells transfected with mRNA encoding tBid.
