# Supplementary material for: Treatment with mRNA coding for the necroptosis mediator MLKL induces antitumor immunity directed against neo-epitopes
Source: Nat Commun. 2018 Aug 24;9:3417. doi: 10.1038/s41467-018-05979-8 (PMC6109072; doi:10.1038/s41467-018-05979-8)
Supplement: Supplementary file 2 — Peer Review [file 41467_2018_5979_MOESM2_ESM.pdf]

Editorial Note: Parts of this peer review file have been redacted as indicated to maintain the confidentiality of unpublished data.

Reviewers' comments:

Reviewer #1 (Remarks to the Author):

In this manuscript, Saelens and colleagues show that transfection with mRNA encoding for MLKL caused cancer cell necroptosis in vitro and in vivo and triggered anti-tumor T cell responses that could restrict tumor growth in syngeneic models of lung and colon cancer. In addition, induction of necroptosis in primary tumors could effectively prevent lung metastasis following i.v. injection of the same cancer cells. Anti-tumor immunity dependent on Batf3-dependent DCs and type I IFNs. Moreover, this approach could also delay the growth of human tumors injected into mice with humanized immune systems. Interestingly, transfection of mRNA encoding tBID also caused tumor cell apoptosis but did not trigger strong anti-tumor immunity similarly to MLKL mRNA transfection, consistent with necroptosis being a much more immunogenic type of cell death compared to apoptosis.

This study goes beyond earlier studies showing that necroptotic cells trigger anti-tumor immunity, as it provides a means of inducing nectroptosis in tumors in vivo, namely by transfection of MLKL mRNA, which is sufficient to trigger the activation of anti-tumor T cell responses. The experiments in humanized mice also support that the approach could function in humans. Overall, these are very interesting results that have potentially important implications for cancer immunotherapy.

Specific comments

It is interesting that transfection of cells with mRNA expressing wild type MLKL caused considerable cell death. Previous studies reported that expression of full length wild type MLKL could not kill cells, in contrast to 'active' MLKL versions that readily induced necroptosis (e.g. Murphy et al, DOI: 10.1016/j.immuni.2013.06.018). Can the authors comment on this discrepancy? What levels of MLKL expression are achieved by this approach compared to endogenous MLKL or MLKL expressed by plasmid transfection? Immunoblot analysis of MLKL expression in cancer cells transfected with mRNA in vitro or in vivo would be helpful to present. It would also be interesting to show if MLKL is phosphorylated in these cells. Would transfection of mRNA encoding for constitutively active MLKL mutants be capable of inducing more efficient cell death and have an even stronger anti-tumor effect?

Matthew Albert and colleagues showed in their 2015 paper in Science that chemically induced RIPK3 dimerisation could trigger immunogenic cell death and anti-tumor immunity, but in this case the production of inflammatory cytokines by the dying cells was required for immunogenicity. In particular, this study claimed that RIPK1-mediated activation of NF-kB-dependent cytokine expression was necessary for CD8 cell cross-priming and anti-tumor immunity following RIPK3 dimerisation-induced cell death. In the current study, cell death is induced by expression of MLKL, which should not activate RIPK1 and NF-kB. Could the authors address if cytokines are expressed in the cells transfected with wild type MLKL mRNA and if cytokine production is required for anti-tumor immunity as in the study by Albert and colleagues? This information will be very important for understanding the mechanisms of necroptosis-induced anti-tumor immunity.

Reviewer #2 (Remarks to the Author):

Hoecke et al. describe that intratumoral administration of mRNA encoding for the necroptosis executioner protein Mlkl leads to significantly delayed tumor growth, both compared to untreated

controls and those tumors injected with mRNA encoding for the apoptosis executioner tBid. In a neoadjuvant and lung colonization study, mice treated with Mlkl mRNA were also resistant to secondary tumor growth. This phenotype was traced to expanded activation of anti-tumor T cells, which did not occur in mice lacking cross-presenting DCs, migratory DCs, or a type I interferon response, suggesting that Mlkl mRNA is inducing immunogenic cell death via necroptosis. The study by Hoecke et al. is of potential interest due to their ability to translate findings regarding necroptosis into a therapeutic treatment. That said there is no mechanistic insight provided beyond the established literature, and there are some discrepancies with the literature that need to be addressed. The therapeutic relevance is also minimized by the failure to compare the approach to established treatments. Specific points are listed below.

Major points:

1) There are opposing results regarding the ability of wt Mlkl overexpression to induce cell death, with possible cell line specific differences (Zhao et al, 2012; Dondelinger et al., 2014; Hildebrand et al., 2014, PNAS; Huang et al., 2017 Mol Cell Biol.; Murphy J. et al., 2013). The statement that these findings are broadly applicable should be tempered. It is also unclear how Mlkl is inducing cell death in the absence of RIPK3 in the CT26 cell line. As the authors describe, Mlkl phosphorylation is usually thought to be required. Do the authors observe phosphorylation and association with the cytoplasmic membrane?

2) The current literature indicates that NF-kB activation during necroptosis is required for immunogenic cell death and the the induction of a DC/CD8+ T cell-mediated anti-tumor response (Yatim et al., Science 2015; Aaes et al, Cell Reports 2016; Yang et al., Oncoimmunology 2016). Given that it was shown RIPK1/RIPK3 RHIM domain dependent interaction mediate NFkB activation, but Mlkl activation is downstream of this event and has not been shown to activate NF-kB in the dying cell, it needs to be shown whether the immunogenicity induced by Mlkl mRNA is an active process requiring transcription or is simply a passive process of cell rupture comparable to necrosis.

3) Apoptosis is generally considered to be nonimmunogenic compared to all other forms of cell death, and the differences shown in the manuscript have been shown elsewhere using artificial systems. Importantly however, many therapies can induce immunogenic cell death and are standard-of-care that the current therapy would need to be compared against. The authors should demonstrate the Mlkl mRNA is superior to radiation, chemotherapy, or simple temperature shock to establish that their approach offers a unique benefit. Ideally this experiment would be done in a therapeutic setting, but even in vitro induction and a vaccination approach would strengthen the clinical relevance of the manuscript.

4) It is nice that they authors demonstrate their therapy requires DCs and the type I IFN receptor to activation OT-I T cells, but this is not equivalent to demonstrating a lack of efficacy in terms of tumor growth.

5) It is unclear why the authors needed to i.v. inject tumor cells to measure metastasis in Figure 5, as this approach creates an experiment that is similar to Figure 4 with the subcutaneous rechallenge, and both the B16 and CT26 models are spontaneously metastatic. The ability to treat established metastatic lesions would be important to demonstrate and more therapeutically relevant.

6) The authors indicate in their discussion that combination studies with Mlkl and checkpoint blockade are likely to show combinatorial efficacy. This should be included in the manuscript for at least PD-1 blockade.

7) In Figure 5 the tBid treated mice show equivalent protection to the Mlkl treated mice. This suggests an equivalent induction of anti-tumor immunity and runs counter to the conclusion that

Mlkl is a superior approach.

8) The human xenograft model in Figure 8 compares MLKL to luciferase, but lacks the tBid control found throughout the rest of the manuscript. It is not possible to conclude from this result that the temporary delay in tumor growth is due to necroptosis versus simple cell death. Engraftment and a characterization of the human immune system in these mice should be shown in the supplemental data.

9) Many experiments do not indicate the number of animals used per group or the number of experimental repeats. This gives the impression that these experiments were done only once (e.g. Figure 2, Figure 3C, 4C, 5C, 6D-F, Figure 7, 8).

Minor points

1) Figure 2D shows cell death only within tumor cells after injections. It would be nice to determine whether stromal cell types are also impacted.

2) There is no data showing protein expression of tBid or MLKL upon treatment in vivo or in vitro.

3) An overlay of tumor growth curves in the supplemental figures would be welcome. Otherwise it is difficult to view small shifts in tumor growth.

4) The histograms in Figure 6 and 7 should be shown flat. Otherwise it is difficult to visualize the differences between the groups.

5) Figure 1 represents a summary of the data and should be at the end of the manuscript.

6) Statistical tests are missing from Figure 7

7) All box plots should be replaced with dot plots (Figure 5, 7 8).

Response to Reviewers' comments:

First, we would like to thank both reviewers for their time, remarks, and concerns about our manuscript. Below, please find our point-by-point responses to the questions that were raised on the work reported in our initial submission (NCOMMS-18-01404).

Reviewer #1 (Remarks to the Author):

In this manuscript, Saelens and colleagues show that transfection with mRNA encoding for MLKL caused cancer cell necroptosis in vitro and in vivo and triggered antitumor T cell responses that could restrict tumor growth in syngeneic models of lung and colon cancer. In addition, induction of necroptosis in primary tumors could effectively prevent lung metastasis following i.v. injection of the same cancer cells. Antitumor immunity dependent on Batf3-dependent DCs and type I IFNs. Moreover, this approach could also delay the growth of human tumors injected into mice with humanized immune systems. Interestingly, transfection of mRNA encoding tBID also caused tumor cell apoptosis but did not trigger strong antitumor immunity similarly to MLKL mRNA transfection, consistent with necroptosis being a much more immunogenic type of cell death compared to apoptosis.

This study goes beyond earlier studies showing that necroptotic cells trigger antitumor immunity, as it provides a means of inducing nectroptosis in tumors in vivo, namely by transfection of MLKL mRNA, which is sufficient to trigger the activation of antitumor T cell responses. The experiments in humanized mice also support that the approach could function in humans. Overall, these are very interesting results that have potentially important implications for cancer immunotherapy.

Specific comments

1) *It is interesting that transfection of cells with mRNA expressing wild type MLKL caused considerable cell death. Previous studies reported that expression of full length wild type MLKL could not kill cells, in contrast to 'active' MLKL versions that readily induced necroptosis (e.g. Murphy et al, DOI: 10.1016/j.immuni.2013.06.018). Can the authors comment on this discrepancy?*

Author response:

It is indeed interesting and at the same time puzzling that transfection of mRNA encoding wild type MLKL can kill cells, although mouse MLKL reportedly is more cytotoxic than human MLKL is (Tanzer et al., CDD, 2016; reference 27 in our manuscript). We have added the following in the discussion section (line 323-327) to address this apparent discrepancy with the literature:

"It is surprising that transfected mRNA encoding wild type MLKL could kill cells because it has been reported that induced expression of wild type MLKL in mouse dermal fibroblasts failed to do so.[22] We speculate that the MLKL expression levels in mRNA transfected cells rapidly reaches a critical concentration that could lead to the formation of amyloid-like polymers that induce necroptosis.[40]"

What levels of MLKL expression are achieved by this approach compared to endogenous MLKL or MLKL expressed by plasmid transfection? Immunoblot analysis of MLKL expression in cancer cells transfected with mRNA in vitro or in vivo would be helpful to present.

Author response:

We have performed immunoblot analysis of B16 melanoma cells and failed to detect endogenous MLKL in these cells with this method. However, Western blot analysis of lysates of MLKL mRNA transfected B16 cells revealed a clear MLKL-specific band. We also monitored the expression and activation status of caspase-3 by Western blot and found caspase-3 processing only in cell lysates of tBid mRNA transfected cells. This result is included as new figure panel 1C in the revised manuscript. The accompanying text was adapted as follows (line 94-100):

"Transfection of B16 cells with tBid-mRNA elicited hallmarks of apoptotic cell death: caspase activity was induced and typical cleavage fragments of caspase-3 were observed, cell death was prevented by the pan-caspase inhibitor zVAD-fmk, and finally cells became sytox positive due to loss of plasmamembrane integrity following secondary necrosis (Figure 1A-C and Figure S2). In contrast, cell death following MLKL-mRNA transfection proceeded without caspase activity or caspase-3 processing, the cells became sytox positive but, in contrast to apoptosis the addition of zVAD-fmk did not prevent cell death, all of which are features of necroptosis (Figure 1A-C and Figure S2)."

It would also be interesting to show if MLKL is phosphorylated in these cells. Would transfection of mRNA encoding for constitutively active MLKL mutants be capable of inducing more efficient cell death and have an even stronger antitumor effect?

Author response:

This is an interesting remark. We analyzed the phosphorylation status of MLKL in mRNA transfected B16 melanoma cells. As a positive control for necroptosis induction we included lysates of L929sAhFas cells that had been stimulated with TNF for 8 hours. MLKL was detectable in lysates of B16 cells that had been transfected with MLKL mRNA and in lysates of L929sAhFas cells. Phosphorylated MLKL, however, was only detectable in the TNF-stimulated L929sAhFas cell lysates (Figure R1).

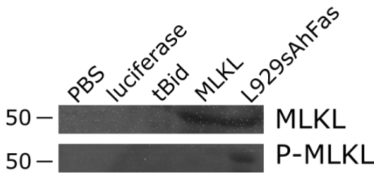

Figure R1. Transfection of MLKL encoding mRNA in B16 cells does not induce detectable phosphorylation of the MLKL protein. One million B16 cells were transfected with PBS or with 1 µg of mRNA encoding luciferase, tBid or MLKL. Twenty four hours after transfection, MLKL and phosphorylated MLKL expression were analyzed in the cell lysates using western blotting. As a positive control for phosphorylation of MLKL, L929sAhFas cells were stimulated with TNF during 8 hours and cell lysates were analyzed by western blotting using anti-MLKL (Millipore, MABC604) and anti-phospho-MLKL antibodies (Abcam; an196436).

Would transfection of mRNA encoding for constitutively active MLKL mutants be capable of inducing more efficient cell death and have an even stronger antitumor effect?

Author response:

To address this, we transfected B16 melanoma cells with mRNA coding for luciferase, tBid, MLKL and a constitutively active mutant of MLKL (MLKLS345D)[1]. Twenty four hours after transfection, cell

viability was monitored by flow cytometry and the percentage of sytox blue positive cells was determined (Figure R2). Transfection with caMLKL mRNA was associated with an increased percentage of cells that became sytox positive compared to tBid and MLKL mRNA transfected cells (approximately 30% compared with approximately 20% for tBid or MLKL mRNA)(Figure R2).

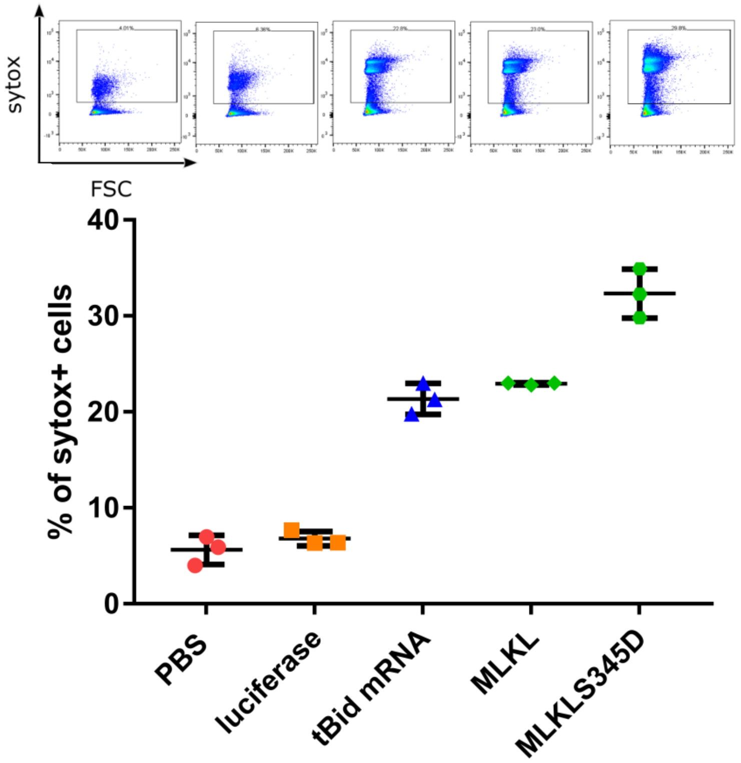

Figure R2. Transfection of mRNA encoding a constitutively active mutant of MLKL results in increased cell death. One million B16 cells were transfected with PBS or with 1 µg of mRNA encoding luciferase, tBid, MLKL or MLKLS345D. Twenty four hours after transfection, cell death was monitored by flow cytometry based on sytox blue uptake. Data points represent the percentage of sytox positive cells in the total population of cells. Horizontal lines represent the mean and standard deviation (SD). The insets above the graph are representative flow cytometry plots with forward scatter (FSC) scaled linearly in the X axis and the sytox blue fluorescence scaled logarithmically in the Y axis. There was no statistically significant difference between the MLKL and MLKLS345D groups.

[Redacted]

2) *Matthew Albert and colleagues showed in their 2015 paper in Science that chemically induced RIPK3 dimerisation could trigger immunogenic cell death and antitumor immunity, but in this case the production of inflammatory cytokines by the dying cells was required for immunogenicity. In particular, this study claimed that RIPK1-mediated activation of NF-kB-dependent cytokine expression was necessary for CD8 cell cross-priming and antitumor immunity following RIPK3 dimerisation-induced cell death. In the current study, cell death is induced by expression of MLKL, which should not activate RIPK1 and NF-kB. Could the authors address if cytokines are expressed in the cells transfected with wild type MLKL mRNA and if cytokine production is required for antitumor*

immunity as in the study by Albert and colleagues? This information will be very important for understanding the mechanisms of necroptosis-induced antitumor immunity.

Author response:

This is indeed an important point. We have analyzed whether MLKL mRNA transfection results in NF-kB activation and found that this is not the case. B16 melanoma cells were transfected with mRNA coding for an irrelevant protein (i.e. GFP), tBid or MLKL together with an NF-kB reporter construct. As positive controls, reporter construct transfected B16 cells were treated with TNF or transfected with a TRAF6 expression vector (known to induce NF-kB). The results of this experiment are included as new figure panel 1D in the revised manuscript and show that MLKL mRNA transfection does not lead to NF-kB induction. This result suggests that NF-kB dependent cytokine expression is not required for the antitumor activity in our models.

The text in the Results section that accords with this new figure panel 1D can be found in line 101-102 and is as follows:

"Cell death induced by mRNA encoding tBid or MLKL did not activate NF-κB signaling in the B16 melanoma cells (Figure 1D)."

In the discussion we added (line 295-299):

"It has been reported that chemically induced RIPK3 dimerisation can trigger immunogenic cell death and anti-tumor immunity by a process that requires NF-κB-dependent cytokine expression and the production of inflammatory cytokines by the dying tumor cells.[17] However, we found no evidence that NF-κB was activated following MLKL mRNA transfection."

Reviewer #2 (Remarks to the Author):

Hoecke et al. describe that intratumoral administration of mRNA encoding for the necroptosis executioner protein Mlkl leads to significantly delayed tumor growth, both compared to untreated controls and those tumors injected with mRNA encoding for the apoptosis executioner tBid. In a neoadjuvant and lung colonization study, mice treated with Mlkl mRNA were also resistant to secondary tumor growth. This phenotype was traced to expanded activation of antitumor T cells, which did not occur in mice lacking cross-presenting DCs, migratory DCs, or a type I interferon response, suggesting that Mlkl mRNA is inducing immunogenic cell death via necroptosis. The study by Hoecke et al. is of potential interest due to their ability to translate findings regarding necroptosis into a therapeutic treatment. That said there is no mechanistic insight provided beyond the established literature, and there are some discrepancies with the literature that need to be addressed. The therapeutic relevance is also minimized by the failure to compare the approach to established treatments. Specific points are listed below.

Major points:

1) *There are opposing results regarding the ability of wt Mlkl overexpression to induce cell death, with possible cell line specific differences (Zhao et al, 2012; Dondelinger et al., 2014; Hildebrand et*

al., 2014, PNAS; Huang et al., 2017 Mol Cell Biol.; Murphy J. et al., 2013). The statement that these findings are broadly applicable should be tempered. It is also unclear how Mlkl is inducing cell death in the absence of RIPK3 in the CT26 cell line. As the authors describe, Mlkl phosphorylation is usually thought to be required. Do the authors observe phosphorylation and association with the cytoplasmic membrane?

Author response:

We have removed “potentially broadly applicable” and “as a broadly applicable intervention” from the discussion.

To help clarify how MLKL induces cell death, we have now documented the expression levels of MLKL in mRNA transfected B16 cells, which shows that these are substantially higher than the endogenous levels (Figure 1C in the revised manuscript). We therefore speculate that high MLKL levels promote the formation of amyloid-like MLKL polymers that can be formed in the absence of RIPK3. This is mentioned as follows in the discussion (line 324-328):

“It is surprising that transfected mRNA encoding wild type MLKL could kill cells because it has been reported that induced expression of wild type MLKL in mouse dermal fibroblasts failed to do so.[22] We speculate that the MLKL expression levels in mRNA transfected cells rapidly reaches a critical concentration that could lead to the formation of amyloid-like polymers that induce necroptosis.[40]”

We analyzed the phosphorylation status of MLKL in mRNA transfected B16 melanoma cells. As a positive control for necroptosis induction we included lysates of L929sAhFas cells that had been stimulated with TNF for 8 hours. MLKL was detectable in the L929sAhFas and B16 cells that had been transfected with MLKL mRNA. Phosphorylated MLKL, however, was only detectable in the TNF stimulated L929sAhFas cell lysates (Figure R1).

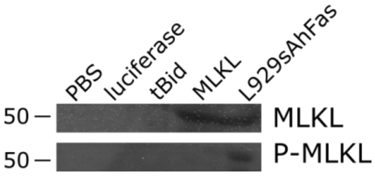

Figure R1. Transfection of MLKL encoding mRNA in B16 cells does not induce phosphorylation of the MLKL protein. One million B16 cells were transfected with PBS or with 1 µg of mRNA encoding luciferase, tBid or MLKL. Twenty four hours after transfection, MLKL and phosphorylated MLKL expression were analyzed in the cell lysates using western blotting. As a positive control for phosphorylation of MLKL, L929sAhFas cells were stimulated with TNF during 8 hours and cell lysates were analyzed by western blotting using anti-MLKL (Millipore, MABC604) and anti-phospho-MLKL antibodies (Abcam; an196436).

2) *The current literature indicates that NF-kB activation during necroptosis is required for immunogenic cell death and the induction of a DC/CD8+ T cell-mediated antitumor response (Yatim et al., Science 2015; Aaes et al, Cell Reports 2016; Yang et al., Oncoimmunology 2016). Given that it was shown RIPK1/RIPK3 RHIM domain dependent interaction mediate NFkB activation, but Mlkl activation is downstream of this event and has not been shown to activate NF-kB in the dying cell, it needs to be shown whether the immunogenicity induced by Mlkl mRNA is an active process requiring transcription or is simply a passive process of cell rupture comparable to necrosis.*

Author response:

This is a very interesting point that we have addressed in the revised manuscript. First, we have analyzed the extend of cell death after mRNA transfection in the presence or absence of actinomycin D. We found that cell death associated with MLKL mRNA transfection of B16 cells was the same in the presence or absence of actinomycin D whereas slightly less cell death was observed in tBid mRNA transfected cells when actinomycin D was added. As a technical control that actinomycin D had worked, the cells were co-transfected with a GFP expression plasmid; actinomycin D treatment abolished GFP expression (Figure 1E in the revised manuscript).

Secondly, we have analyzed whether MLKL mRNA transfection results in NF-kB activation. This was not the case. B16 melanoma cells were transfected with mRNA coding for an irrelevant protein (i.e. GFP), tBid or MLKL together with an NF-kB reporter construct. As positive controls, reporter construct transfected B16 cells were treated with TNF or transfected with a TRAF6 expression vector, which is known to induce NF-kB. MLKL mRNA transfection does not lead to NF-kB induction. This result suggests that NF-kB dependent cytokine expression is not required for the antitumor activity in our models. The results of this experiment are included as new figure panel 1D in the revised manuscript.

This is mentioned as follows in the results (line 101-105)

"Cell death induced by mRNA encoding tBid or MLKL did not activate NF-κB signaling in the B16 melanoma cells (Figure 1D). Moreover, the extend of cell death following tBid or MLKL mRNA transfection was comparable in the presence or absence of actinomycin D, suggesting that *de novo* transcription was not required (Figure 1E)."

3) *Apoptosis is generally considered to be nonimmunogenic compared to all other forms of cell death, and the differences shown in the manuscript have been shown elsewhere using artificial systems. Importantly however, many therapies can induce immunogenic cell death and are standard-of-care that the current therapy would need to be compared against. The authors should demonstrate the Mlkl mRNA is superior to radiation, chemotherapy, or simple temperature shock to establish that their approach offers a unique benefit. Ideally this experiment would be done in a therapeutic setting, but even in vitro induction and a vaccination approach would strengthen the clinical relevance of the manuscript.*

Author response:

This is a very interesting remark that we have addressed in a new in vivo therapeutic experiment using C57/Bl6 mice that had been inoculated subcutaneously with B16 melanoma cells. We focused on a chemotherapeutic regimen with doxorubicine (dox) for comparison with the mRNA treatments. We compared 7 different groups (8 mice/group): PBS treated, luciferase mRNA, tBid mRNA, MLKL mRNA, dox (3 mg/kg) injected intraperitoneally every second day during 3 weeks, dox administered intratumorally every second day during 3 weeks and dox administered intratumorally on days 6, 8 and 10 after tumor inoculation. MLKL mRNA treatment resulted in significantly longer survival of the mice compared with any of the dox treatment set ups. In addition, the prolonged treatment with dox during 3 weeks was associated with significantly reduced body weight loss and lymphocytopenia, compared with the MLKL mRNA treated group. Thus, MLKL mRNA tumor

treatment prolongs mouse survival significantly better than dox treatment (administered i.p. or i.t.). Moreover, the prolonged treatment with dox, which results in significantly longer mouse survival compared with the negative control treated mice, is associated with significant body weight loss and lymphocytopenia. These results are included in the revised manuscript as a new figure 3. In lines 139-148 of the revised manuscript, we added the following:

"Some of the standard of care therapies for cancer patients can induce immunogenic cell death, such as treatment with doxorubicin (dox).[12] We compared the antitumor effect of the MLKL-mRNA treatment approach with repeated injections of dox in the B16 melanoma model (Figure 3A). Dox injections were administered every other day into the tumor or intraperitoneally for 3 weeks. Mice from another group were treated on day 6, 8 and 10 only, by intra-tumor injection of dox. MLKL-mRNA treatment was associated with significantly prolonged survival of the mice compared with any of the dox treatment set ups (Figure 3B, 3C and Figure S3). In addition, the prolonged treatment with dox during 3 weeks was associated with significantly reduced body weight loss and lymphocytopenia compared with the MLKL-mRNA treated group (Figure 3D and 3E)."

4) *It is nice that they authors demonstrate their therapy requires DCs and the type I IFN receptor to activation OT-I T cells, but this is not equivalent to demonstrating a lack of efficacy in terms of tumor growth.*

Author response:

We agree with this remark. The CD8-depletion experiment shown in figure 7D (Figure 5D in the original submission) shows that the antitumor effect of MLKL mRNA treatment depends on CD8+ T cells. The in vivo cell killing and OT-I proliferation experiments shown in figure 8 (Figure 6 in the original submission) show that CD8 T cell activation and effector function are strongly reduced after MLKL mRNA tumor treatment in the type IFN receptor, Batf3 and CCR7 knock out mice. Together these data suggest that the antitumor activity that can be induced with MLKL mRNA will likely be strongly blunted in IFNR1, Batf3 and CCR7 deficient mice. Nevertheless we have weakened our statement in this context by changing line 236 as follows: "Antitumor T cell immunity following MLKL-mRNA treatment depend on migratory Batf3 DCs and type I IFN signaling"

is now:

"Antitumor T cell responses following MLKL-mRNA treatment depend on migratory Batf3 DCs and type I IFN signaling"

5) *It is unclear why the authors needed to i.v. inject tumor cells to measure metastasis in Figure 5, as this approach creates an experiment that is similar to Figure 4 with the subcutaneous rechallenge, and both the B16 and CT26 models are spontaneously metastatic. The ability to treat established metastatic lesions would be important to demonstrate and more therapeutically relevant.*

Author response:

We specifically opted for the lung colonization assay with intravenous injection of tumor cells to analyze the effects of the mRNA treatment strategy on the systemic outgrowth of melanoma and CT26 cells at distant sites. Indeed, our main objective was to analyze whether intratumoral delivery of MLKL mRNA in the primary tumor might hamper the outgrowth of tumor cells at distant sites by a systemic antitumor immune response rather than by blocking the metastatic cascade. As such, we believe that our approach is therapeutically relevant and representative for patients in which tumor cells have already spread towards distant sites at diagnosis, as is the case for many aggressive cancer types such as melanoma.

To exclude potential effects on the invasive and metastatic capacities of the primary tumor, equal number of cells were injected in the tail vein in all conditions. As primary tumor growth is affected by the treatment strategy, the number of metastatic lesions might be skewed between conditions due to primary tumor modulation rather than a systemic antitumor immune response. To avoid confusion regarding this matter, we renamed the "metastasis assay" as "lung colonization assay" and metastases are defined as pulmonary colonies therein.

Additionally, the reviewer refers to the intrinsic metastatic capacities of B16 and CT26 tumor models. However, it should be noted that for both lines, in particular for the B16 melanoma cells, the metastatic abilities differ between labs, as many clones and sublines of the cellular tumor models are used worldwide.

Finally, to address the point that an established lesion could also be affected by treatment of a tumor at another site, we opted to use a model in which tumor cells were inoculated at both sides of the mouse before mRNA treatment was started. Importantly, only one of the tumors was treated, and the growth of the other one was monitored over time. This model can be considered as a surrogate for a pre-existing metastatic lesion.[2] tBid mRNA treatment of the tumor that was inoculated first was associated with a slightly reduced growth rate of the second tumor. This abscopal effect was, however, much more pronounced after MLKL mRNA treatment of the first tumor. The results of this experiment are included in the revised manuscript as new Figure panel 4D accompanied with the following text (lines 165-171):

"We also tested the systemic immunity in a second model where a possible abscopal effect could be evaluated.[42] Mice were inoculated with B16 cells in either flank but on different days: the tumor in the left flank was implanted three days later than the tumor in the right flank. Only the tumor in the right flank of the animals was subsequently treated, starting at day 6 after the first inoculation. The growth of the distant untreated tumor in the left flank was monitored over time (Figure 4D). Also in this set-up a pronounced delay in tumor growth of the untreated tumor was observed in the case of MLKL-mRNA treatment (Figure 4D and Figure S3)."

6) *The authors indicate in their discussion that combination studies with Mlkl and checkpoint blockade are likely to show combinatorial efficacy. This should be included in the manuscript for at least PD-1 blockade.*

<u>Author response</u>:

This is a very good suggestion. We have analyzed the antitumor effect of MLKL mRNA treatment combined with a PD1 inhibitory or an isotype control antibody. This experiment was performed with

C57Bl/6 mice that had been inoculated with B16 cells on day 0 in one flank and 3 days later in the opposite flank. Only the tumor that was inoculated first was treated and growth of both the treated and untreated tumors was monitored over time. We found that anti-PD1 treatment (intraperitoneal injection starting on day 6, every 3 days for 3 weeks) combined with PBS or luciferase mRNA treatment of the tumor that was inoculated first was associated with a slight delay in growth of the B16 tumors on either side of the mice compared with the isotype control antibody treatment. MLKL mRNA tumor treatment strongly suppressed growth of both tumors, and, when combined with anti-PD1 treatment prevented growth of either tumor. This finding shows that anti-PD1 treatment combined with MLKL mRNA treatment has a superior antitumor effect compared with either treatment alone. The results of this experiment have been added to the revised manuscript as a new figure 6. The accompanying text is as follows (line 183-199):

"**Combining MLKL-mRNA treatment with PD1 blockage improves the antitumor effect**

The antitumor activity of MLKL-mRNA treatment might be further improved when combined with cancer treatment options that are already clinically established such as checkpoint blockade approaches. Once inside the tumor bed, T cells primed by intratumor MLKL-mRNA treatment might be silenced by multiple immune suppressive mechanisms used by tumors to evade elimination.[43] Checkpoint inhibitors such as anti-CTLA4, -PD-1 and -PD-L1, IDO inhibitors or Treg depletion strategies primarily act by taking away these breaks. However, these treatments are unfortunately poorly effective in patients with tumors with a low number of tumor-infiltrating T cells.[6] Since the MLKL-based mRNA therapy reported here induces robust infiltration of APCs into the tumor, it is possible that a combination therapy with a checkpoint inhibitor could further improve the curative potential of intratumor delivery of MLKL-mRNA. B16 tumors were implanted s.c. in the right flank of the mice and three days later in the left flank of the mice. On day 6, the tumor that was inoculated first was treated with MLKL mRNA in combination with i.p. administration of anti-PD-1 or an isotype control antibody (Figure 6A). The anti-PD1 combination therapy was significantly more effective at suppressing the growth of the primary treated tumor and of the distant untreated tumor than the MLKL-mRNA treatment combined with the control antibody (Figure 6B, 6C and Figure S3)."

We have also included this finding in the abstract as follows:

"Moreover, combining the MLKL-mRNA treatment with immune checkpoint blockade further improves the antitumor activity."

7) *In Figure 5 the tBid treated mice show equivalent protection to the Mlkl treated mice. This suggests an equivalent induction of antitumor immunity and runs counter to the conclusion that Mlkl is a superior approach.*

Author response:

This is correct. The limitation of the experiment that was (and still is) shown is that the ethical endpoint for the PBS and luciferase treated mice was reached on day 26 after the start of the experiment. Therefore, we sacrificed the tBid and MLKL mRNA treated mice on the same day for comparison. We have now repeated the experiment with i.v. injected B16 F10 melanoma cells using 8 mice per group for the PBS and luciferase mRNA setup and 16 mice per group for the tBid and MLKL mRNA treated groups. All PBS and luciferase mRNA treated mice and half of the mice in the

tBid and MLKL mRNA treated groups were sacrificed on day 26 after the first tumor cell inoculation. As before, we found that tBid and MLKL mRNA treatment resulted in respectively very few and no detectable tumor nodules in the lungs of the mice on day 26. The remaining 8 mice from the tBid and MLKL mRNA treated groups were sacrificed on day 36 after the treatment. At this time point, tBid mRNA treated mice displayed around 200 tumor nodules per lung whereas there were still no nodules visible in the MLKL mRNA treated mice. Therefore, we can conclude that MLKL mRNA treatment is superior to tBid mRNA treatment in this model. These new results are shown in figure 5B of the revised manuscript.

8) *The human xenograft model in Figure 8 compares MLKL to luciferase, but lacks the tBid control found throughout the rest of the manuscript. It is not possible to conclude from this result that the temporary delay in tumor growth is due to necroptosis versus simple cell death. Engraftment and a characterization of the human immune system in these mice should be shown in the supplemental data.*

Author response:

We have repeated the experiment in the humanized mouse model and now included human tBid mRNA treatment for comparison. In this new experiment, tBid mRNA treatment performed slightly better than luciferase mRNA in controlling tumor growth. However, the strongest restriction of the tumor growth was observed with human MLKL mRNA treatment. Unfortunately, we lost several mice in the course of the experiment (1 in the PBS and luciferase mRNA groups and 3 in the tBid and MLKL mRNA groups), presumably due to graft versus host activity. These new data are shown in the new supplementary figure 9 in the revised manuscript.

The engraftment and characterization of the human system in the NGS mice are now shown in Supplementary Figures S7 and S8 and in table S1.

9) *Many experiments do not indicate the number of animals used per group or the number of experimental repeats. This gives the impression that these experiments were done only once (e.g. Figure 2, Figure 3C, 4C, 5C, 6D-F, Figure 7, 8).*

Author response:

We apologize that this information was unclear in the original manuscript. The number of animals and repeats have now been added in the Figure legends.

Minor points

1) Figure 2D shows cell death only within tumor cells after injections. It would be nice to determine whether stromal cell types are also impacted.

Author response:

We agree with this suggestion. However, the ex vivo flow cytometry analysis for cell death is technically very challenging due to the difficulty to separate the individual cells from the tissue.

2) There is no data showing protein expression of tBid or MLKL upon treatment in vivo or in vitro.

Author response:

We have now documented caspase-3 processing in tBid mRNA transfected cells and MLKL expression in MLKL mRNA transfected cells in vitro in figure 1C.

3) An overlay of tumor growth curves in the supplemental figures would be welcome. Otherwise it is difficult to view small shifts in tumor growth.

Author response:

Such an overlay figure has been added to the manuscript as supplemental Figure S3.

4) The histograms in Figure 6 and 7 should be shown flat. Otherwise it is difficult to visualize the differences between the groups.

Author response:

The figures have been adapted accordingly.

5) Figure 1 represents a summary of the data and should be at the end of the manuscript.

Author response:

We agree with this suggestion and have moved this figure at the end of the manuscript and is now Figure 10.

6) Statistical tests are missing from Figure 7

Author response:

Statistical testing has now been added to Figure 7.

7) All box plots should be replaced with dot plots.

Author response:

This has been corrected in the revised manuscript.

References.

1 Murphy, J. M. *et al.* The pseudokinase MLKL mediates necroptosis via a molecular switch mechanism. *Immunity* **39**, 443-453, doi:10.1016/j.immuni.2013.06.018 (2013).

2 Singh, M. *et al.* Intratumoral CD40 activation and checkpoint blockade induces T cell-mediated eradication of melanoma in the brain. *Nature communications* **8**, 1447, doi:10.1038/s41467-017-01572-7 (2017).

REVIEWERS' COMMENTS:

Reviewer #1 (Remarks to the Author):

The authors have addressed my concerns in the revised manuscript. I recommend publication of the paper.

Reviewer #2 (Remarks to the Author):

Hoecke et al. have done an excellent job addressing the concerns of the reviewers. The only major issue remaining is that several new experiments have been performed only once (Figure 4D, 6, 9). These need to be repeated prior to publication. When repeating Figure 6 the tBID control would be helpful to include, at least in supplemental data. The tBID control could replace the use of the PBS control to keep the experiment manageable. Also note that there are currently two Figure 3D panels.

Response to reviewers

We thank the reviewers for their time and supportive comments to our revised manuscript.

<u>Reviewer #1 (Remarks to the Author):</u>

The authors have addressed my concerns in the revised manuscript. I recommend publication of the paper.

<u>Author response</u>:

Very much appreciated.

<u>Reviewer #2 (Remarks to the Author):</u>

Hoecke et al. have done an excellent job addressing the concerns of the reviewers.

<u>Author response</u>:

Very much appreciated.

The only major issue remaining is that several new experiments have been performed only once (Figure 4D, 6, 9). These need to be repeated prior to publication. When repeating Figure 6 the tBID control would be helpful to include, at least in supplemental data. The tBID control could replace the use of the PBS control to keep the experiment manageable.

<u>Author response</u>:

We agree that it is important to perform repeat experiments. We felt under time pressure during the revision period and, therefore, could not always organize a repeat of all the experiments that were performed for the revised manuscript. However, the negative controls and MLKL mRNA groups in Figure 4D and 9 have been repeated elsewhere in the manuscript.

Figure 4D is based on data that were generated during the revision period and shows that MLKL mRNA treatment of a tumor (also) slows down the growth of an untreated tumor that was inoculated in the opposite flank of the mice, 3 days before the treatment was started. PBS and luciferase mRNA treatment groups behave the same: the untreated tumor grows. tBid mRNA somewhat slows down the tumor growth but clearly not to the extent of MLKL mRNA. The PBS, luciferase and MLKL mRNA treatment groups were, in fact, repeated in the experiment shown in Figure 6C. Figure 6, also generated based on an experiment that was performed during the revision period, shows that anti-PD1 treatment further strengthens the MLKL mRNA anti-tumor effect. In Figure 6C this is shown for an untreated tumor in a model that is the same as in figure 4D. The PBS, luciferase and MLKL + isotype control (instead of anti-PD1) groups can thus be considered a repeat of those same groups shown in figure 4D. The tBid mRNA group was not included in this experiment. However, in figure 4D we intentionally used relatively large groups of mice (8 per group) for the tBid and MLKL mRNA set ups to have more replicates within one experiment.

The data shown in **figure 6**, where we document that anti-PD1 further improves the MLKL mRNA anti-tumor effect, are indeed from a single experiment. However, we used relatively large groups of mice (8 per groups). We did not include a tBid mRNA with anti-PD1 here. The intention of the experiment was to evaluate if MLKL mRNA treatment could be strengthened with check point inhibitor blockade, a possibility that was mentioned in the last paragraph of the discussion of our originally submitted manuscript. We did not intend to evaluate tBid mRNA combined with anti-PD1 and did not perform such an experiment.

The experiment of which the results are shown in **Figure 9**A is largely repeated in Supplementary figure 9A, which was performed in the revisions period. Admittedly, cell death of human tumor cells following human tBid mRNA transfection is only shown in Supplementary Figure 9A. The *in vivo* tumor treatment with PBS, luciferase or MLKL mRNA is shown in Figure 9B and repeated in Supplementary figure 9B. *In vivo* tBid mRNA treatment in this fairly complex and lengthy humanized mouse model, was performed only once, with 6 mice, and the result is shown in Supplementary figure 9B.

We agree that a repeat of the tBid mRNA experiments in figure 4D and Supplementary figure 9 would be ideal. However, although we consistently observed that tBid mRNA treatment was less effective in preventing tumor growth than MLKL mRNA treatment, we do not mention this in the abstract of the manuscript. The main conclusion of this work is, rather, that MLKL mRNA treatment of tumors is an efficient way to induce anti-tumor immunity.

Also note that there are currently two Figure 3D panels.

<u>Author response</u>:

We thank the reviewer for pointing out this error. This has been corrected.
